# Cell-specific clock-controlled gene expression program regulates rhythmic fiber cell growth in cotton

**DOI:** 10.1186/s13059-023-02886-0

**Published:** 2023-03-14

**Authors:** Dehe Wang, Xiao Hu, Hanzhe Ye, Yue Wang, Qian Yang, Xiaodong Liang, Zilin Wang, Yifan Zhou, Miaomiao Wen, Xueyan Yuan, Xiaomin Zheng, Wen Ye, Boyu Guo, Mayila Yusuyin, Eugenia Russinova, Yu Zhou, Kun Wang

**Affiliations:** 1 State Key Laboratory of Hybrid Rice, College of Life Sciences, Wuhan University, Wuhan, China; 2 Hubei Hongshan Laboratory, Wuhan, China; 3 Institute for Advanced Studies, Wuhan University, Wuhan, China; 4 TaiKang Center for Life and Medical Sciences, RNA Institute, Remin Hospital, Wuhan University, Wuhan, China; 5 Medical Research Institute, Frontier Science Center for Immunology and Metabolism, School of Medicine, Wuhan University, Wuhan, China; 6 Department of Plant Biotechnology and Bioinformatics, Ghent University, Ghent, Belgium; 7 Center for Plant Systems Biology, VIB, Ghent, Belgium; 8 Research Institute of Economic Crops, Xinjiang Academy of Agricultural Sciences, Urumqi, China

**Keywords:** scRNA-seq, Rhythmic regulation, Fiber development, Cotton

## Abstract

**Background:**

The epidermis of cotton ovule produces fibers, the most important natural cellulose source for the global textile industry. However, the molecular mechanism of fiber cell growth is still poorly understood.

**Results:**

Here, we develop an optimized protoplasting method, and integrate single-cell RNA sequencing (scRNA-seq) and single-cell ATAC sequencing (scATAC-seq) to systematically characterize the cells of the outer integument of ovules from wild type and fuzzless/lintless (*fl*) cotton (Gossypium *hirsutum*). By jointly analyzing the scRNA-seq data from wildtype and fl, we identify five cell populations including the fiber cell type and construct the development trajectory for fiber lineage cells. Interestingly, by time-course diurnal transcriptomic analysis, we demonstrate that the primary growth of fiber cells is a highly regulated circadian rhythmic process. Moreover, we identify a small peptide GhRALF1 that circadian rhythmically controls fiber growth possibly through oscillating auxin signaling and proton pump activity in the plasma membrane. Combining with scATAC-seq, we further identify two cardinal cis-regulatory elements (CREs, TCP motif, and TCP-like motif) which are bound by the *trans factors GhTCP14s* to modulate the circadian rhythmic metabolism of mitochondria and protein translation through regulating approximately one third of genes that are highly expressed in fiber cells.

**Conclusions:**

We uncover a fiber-specific circadian clock-controlled gene expression program in regulating fiber growth. This study unprecedentedly reveals a new route to improve fiber traits by engineering the circadian clock of fiber cells.

**Supplementary Information:**

The online version contains supplementary material available at 10.1186/s13059-023-02886-0.

## Background

Cotton fibers are an important natural cellulose source for the textile industry worldwide [1]. Every fiber is a highly elongated single-celled trichome on the surface of cotton seeds. During the development of cotton ovules to seeds, some epidermal cells from the outer layer of the ovule differentiate into fiber primordial cells (also called fiberblast), and eventually develop into fiber cells, accounting for around 30–50% of epidermal cells dependent on the cotton type [2]. There are two waves of primordial cells bulging out the epidermis surface to initiate elongation. The first wave of cells starts protruding on the day of anthesis or shortly thereafter, during which the cells ultimately elongate to 2–3.5 cm in length as lint fibers, and the second wave of cell protruding occurs at 4–10 days post-anthesis (DPA), during which the cells elongate to 0.1 cm as fuzzy fibers. The lint fibers are spinnable, an important agronomic trait for the textile industry.

The cotton fiber development has been receiving attention from cotton researchers worldwide. To date, genetic studies have identified several genes in controlling the different processes of fiber development. The MYB-type transcription factors (TFs) such as *GhMYB25-like* [3], *GhMYB25* [4], *GaMYB2* [5], and homeodomain-leucine zipper (HD-ZIP) IV TF such as *GhHD-1* [6], control the fiber initiation; cotton brassinosteroid biosynthesis genes, *GhDET2* [7] and *PAG1* [8], and the HD-ZIP TF *GhHOX3* [9], regulate the fiber elongation; a LIM domain protein *WLIM1a* [10] and a NAC TF *FSN1* [11] promote the secondary cell wall formation of fibers. Intriguingly, small RNAs miR828 and miR858 were also found to modulate fiber growth through silencing *GhMYB2* [12]. However, the molecular mechanisms of this spatiotemporally dynamic and highly complex network remain elusive.

The circadian clock functions as a pacemaker in controlling many aspects of plant development and physiology, such as stomatal opening, hypocotyl elongation, flowering time, light responses, and biomass [13]. In crops, the natural allelic variants that change the pace of the clock can enhance the growth performance in tomatoes [14] and soybean [15], supporting the longstanding idea that manipulating the pacemaker (circadian clock) can improve yield and other agricultural traits [16]. In cotton, the circadian cycle has been linked to fatty acid metabolism [17] and ethylene biogenesis [18] in seedlings, both of which are known to regulate fiber development [19]. However, whether there is a direct link between circadian rhythm and fiber growth is unknown yet.

Recent technical advances in high-throughput single-cell RNA sequencing (scRNA-seq), especially the newly developed microfluidic droplet technology, have enabled us to quantify the genome-wide RNA expression at the single-cell resolution for an entire tissue or organ [20]. However, due to the particularity of plant tissues from different species, a customized protocol is often required in protoplast preparation, which limits the application of scRNA-seq. In recent years, the studies of scRNA-seq with droplet technology in plants have been limited to a few plant species and tissues, such as the roots of *Arabidopsis thaliana*, rice, and maize [21–28], leaves of *Arabidopsis thaliana* [29–31], stems of *Populus alba* [32], and the gametophytes of *Physcomitrella patens* (data from 10 × Genomics Inc.). Some researchers have to use protoplasting-free single-nucleus RNA-seq as an alternative technology [33, 34].

To systematically decode the gene regulatory network controlling the growth of cotton lint fiber cells, here we developed a customized protocol named Partial Tissue Enzymatic Digestion (PTED) to prepare protoplasts from out integument (OI) of the ovules at the early developmental stages of lint fiber cells (− 2 to 2 DPA). We performed scRNA-seq and scATAC-seq of the protoplasts to compare the single-cell transcriptome and chromatin accessibility landscapes between an upland cotton (*G. hirsutum*) cultivated species Xuzhou142 (wildtype, WT) and its natural fuzzless/lintless mutant (*fl*). We identified and characterized the fiber cells for the first time, and resolved the developmental trajectory for these lint fiber cells. Surprisingly, we found that the early growth of fiber cells exhibits a significant circadian oscillating pattern, and we further decoded the regulatory mechanism mediated by fiber cell-specific clock-controlled genes (CCGs).

## Results

### PTED protoplasting scRNA-seq for outer integument cells of cotton ovules

**Fig. 1:**
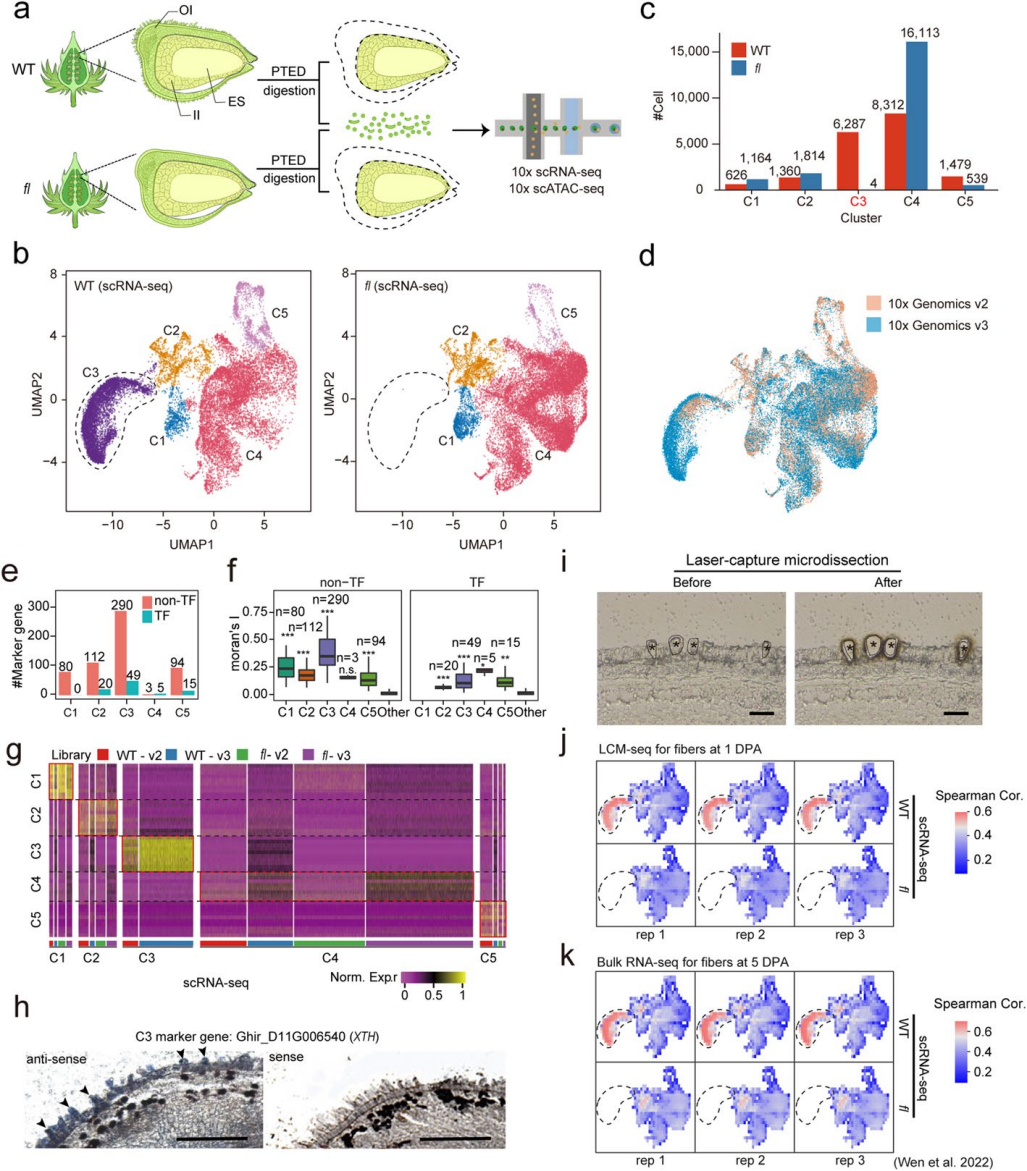
Construction of single-cell transcriptional profile for the outer integument cells of the cotton ovule. **a** Schematic of the experimental design. A development period mixed sample (− 2DPA to 2DPA) of ovule outer integument cells from WT and *fl* were separately enzymatically digested for scRNA-seq and scATAC-seq sequencing. The OI, II, and ES denote the out integument, inner integument, and embryo sac of ovules, respectively. **b** UMAP projection of WT (left) and *fl* (right) scRNA-seq data. Clusters are labeled by color. The lost cell cluster in *fl* is marked with a dotted line circle. **c** Distribution of cell numbers for WT and *fl* scRNA-seq data in each cell cluster. **d** UMAP projection showing that the batch effect difference between data of different platforms is well eliminated. See also Additional files 2 and 3: Table S1-2. **e** The number of marker genes identified by scRNA-seq for transcriptional factors and non-transcriptional factors in each cell cluster. **f** The distribution of Moran’s I score for marker genes and other genes. The number of marker genes in each group and the *p* values from two-sided Wilcoxon tests between marker genes and other genes are shown at the top (* represent *p* < 1E − 3, ** represent *p* < 1E − 6, *** represent *p* < 1E − 9). **g** Heatmap showing normalized gene expression values for top 10 marker genes in each cell cluster. **h** In situ hybridization of XTH gene with RNA probes of antisense (left) and sense (right). The black triangles show the signals from fiber cells, scale bar = 300 μm. **i** A representative example illustrating the capture of fiber cells by laser-capture microdissection (LCM). The fiber cells indicated by star symbols were cut from the epidermis of an ovule at 1 DPA. Scale bar, 50 μm. **j**,**k** The gene expression correlation analysis between the cells in the UMAP and 1 DPA fiber cells of LCM-seq (**j**), and 5 DPA fiber cells of bulk RNA-seq (**k**) in three replicates. The Spearman correlation coefficients are shown for the cells in the UMAP of scRNA-seq from WT (top) and *fl* (bottom). The dotted line circle marks the C3 cell cluster from scRNA-seq

The cotton ovules have two layers of envelopes called outer integument (OI) and inner integument (II), respectively (Fig. 1a). Cotton fiber quality and density are determined by OI, where the fibers initiate [2]. To characterize the cell types in OI of ovules, we performed scRNA-seq for the upland cotton (*Gossypium hirsutum*) cultivar Xuzhou 142 wild type (WT) and its fuzzless/lintless mutant (*fl*), which does not develop to fibers.

We devised a novel protoplasting method named Partial Tissue Eenzymatic Digestion (PTED, see “Methods” for details), which enabled us to directly extract protoplasts from the OI of ovules at the initiation stages of fiber cells (− 2 to 2 DPA) for WT and *fl* (Additional file 1: Fig. S1a) by skipping the dissection step. With PTED, in 1.5 h of treatment, we can obtain enough protoplast cells with a high viability ratio (> 85%, Additional file 1: Fig. S1b). Only the OI layer of the ovules was well digested, while the nucellus and inner integument layers were retained (Additional file 1: Fig. S1c).

Using PTED, we collected the protoplast cells from the OI of ovules (− 2 to 2 DPA) for WT and *fl* respectively. The two groups of protoplast cells were separately used to perform scRNA-seq parallelly (Fig. 1a). For scRNA-seq, we successfully captured 18,064 WT and 21,389 fl cells in two biological replicates with 10 × Genomics v2 and v3 kits, which produced 98,263,514 and 109,914,075 reads, and detected 58,081 and 59,869 genes for WT and *fl* samples, respectively (Additional file 2: Table S1).

### Fiber cells identified in ovule outer integument

We performed dimensionality reduction analysis for the WT and *fl* scRNA-seq data with Uniform Manifold Approximation and Projection (UMAP) [35]. To understand the overall cell distribution, we identified 251 Modularity Enriched Genes (MEGs) expressed at specific locations on the UMAP embedding and discovered that the cells could be split into four groups with MEG expression and one group without MEGs (Additional file 1: Fig. S2a-b). Guided by the location of MEGs on the UMAP embedding, the 12 cell groups from unsupervised clustering were merged into five clusters, C1–C5 (Fig. 1b,c, Additional file 1: Fig. S2c-d). The strong expression correlation between the biological replicates with v2 and v3 kits indicates that our scRNA-seq data are highly reproducible for WT and *fl*, respectively (Additional file 1: Fig. S2e). The batch effects between the two sequencing kits and between WT and *fl* samples in the scRNA-seq data are also well eliminated (Fig. 1d, Additional file 1: Fig. S2f).

To annotate the cell clusters that we found for the cotton ovules OI, we next identified 668 marker genes for the five cell clusters C1–C5, including 89 transcription factors (TFs) and 579 non-TFs (Fig. 1e and Additional file 3: Table S2). The cluster C3 has the largest number of marker genes (49 TFs and 290 non-TFs). The marker genes have high expression specificity among cell clusters, as indicated by Moran’s I scores (Fig. 1f), and shown by the normalized gene expression profiles across single cells grouped by cell clusters (Fig. 1g). The expression patterns of the marker genes are consistent between different sequencing kits and sample types (WT and *fl*). The relative expressions of representative gene examples for C1 to C5 in all single cells are shown on the UMAP embedding for both WT and *fl* samples (Additional file 1: Fig. S3a left), and their average relative expressions in different cell clusters support they are marker genes (Additional file 1: Fig. S3a right).

We found that WT has a unique cell cluster C3 which is missing in *fl* mutant when comparing the two cell cluster clouds (Fig. 1b). Considering that *fl* ovules have a phenotypic deficiency in fiber initiation compared to WT ovules, we inferred that the C3 cluster that is lost in *fl* represents the fiber cell type. This reasoning is further strengthened based on the following pieces of evidence. First, most of top gene ontology terms (GO-terms) enriched in the TF marker genes of the C3 cluster are directly linked to the cell differentiation and development of trichome or seed, and related processes (Additional file 1: Fig. S4). Second, the marker genes in the C3 cluster include multiple known cotton fiber-associated transcription factors: *MYB25* [4, 36], *GhMYB109* [37], *GhHOX3* [9], fiber initials abundantly expressed transcription factor *GhTCP14* [38], and cotton fiber traits-associated genes from GWAS: *GhTUA9* [39] and *qFWPB_D02* [40] (Additional file 1: Fig. S5 and Additional file 3: Table S2). Third, according to the previous literature, the following highly expressed genes in the C3 cluster have been reported to accumulated predominantly in fiber cells with evidence from RNA in situ hybridization, GUS reporter or quantitative RT-PCR assays: *GaMYB2* [5], *GhHD1* [6], *GhFLA1* [41], *GbPDF1* [42], *GhABP19/20* [43], *GhACTIN1* [44] (Additional file 1: Fig. S5 and Additional file 3: Table S2). Furthermore, we confirmed that the representative marker gene *XTH* (*Xyloglucan Endotransglycosylases/Hydrolases*) of C3 cluster (Additional file 1: Fig. S3a) is specifically expressed in fiber cells with in situ hybridization (Fig. 1h).

To further verify that the cells of C3 cluster are fiber cells, we used laser-capture microdissection (LCM) to capture fiber cells at 1 DPA ovule (Fig. 1i) coupled with RNA sequencing (LCM-seq). The LCM-seq data from the three biological replicates exhibit good reproducibility (Additional file 1: Fig. S3b). Importantly, analysis of expression correlation shows that the gene expression profiles of fiber cells from LCM have the highest correlation with C3 cluster (Additional file 1: Fig. S3c). Furthermore, we performed expression correlation analysis between the cells in the UMAP and fiber cells from LCM and found that the cells in C3 cluster have the highest correlations with the fiber cells (Fig. 1j). These results provide convincing evidence supporting the C3 cluster represents cotton fiber cells.

Moreover, we reanalyzed our recently published bulk RNA-seq data from manually separated fiber cells at 5 DPA [45]. The RNA-seq data of the three replicates show great reproducibility (Additional file 1: Fig. S3d). We next compared the gene expression patterns of these fiber cells with that of the five cell clusters (C1–C5), and we found that the gene expression correlation with the C3 cluster is the highest, followed by the clusters C2 and C1 (Additional file 1: Fig. S3e). We also performed expression pattern correlation analysis between the cells in the UMAP and the three replicates of the collected fiber cells, and significantly, the C3 cell cluster shows the highest correlations with all three replicates (Fig. 1k).

In summary, we have identified five major cell types and their marker genes for the OI layer of cotton ovules through integrative analysis of WT and *fl* single-cell RNA-seq data, and recognized a population of fiber cells (C3 cluster) present in WT but absent in *fl* mutant*.*

### Fiber developmental trajectory and determining genes

**Fig. 2:**
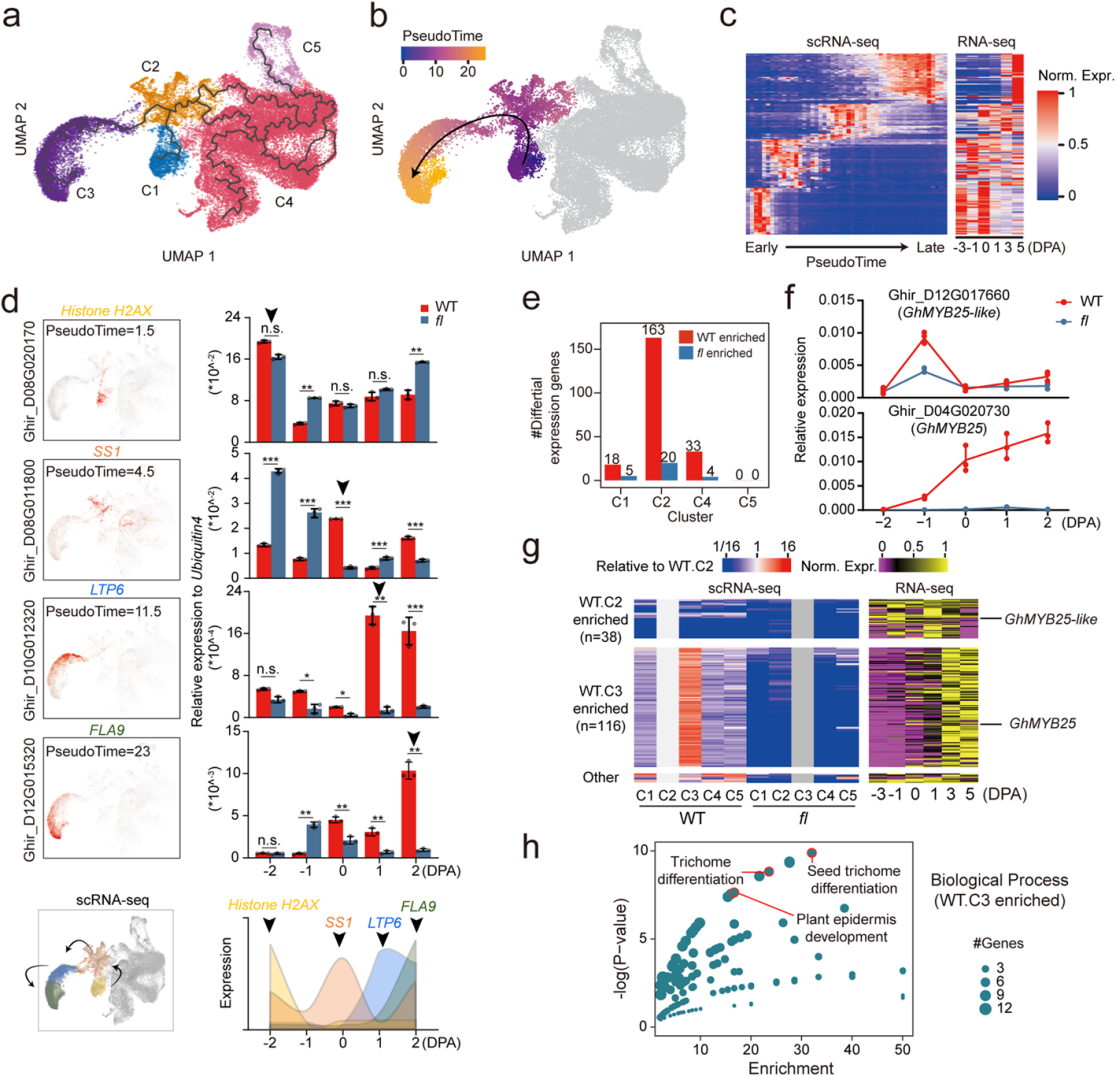
The fiber cells developmental trajectory. **a**,**b** The cell development trajectories (**a**) and pseudo-time for fiber cells (**b**) by using Monocle3. **c** Expression heatmap of scRNA-seq and time-course RNA-seq for highly dynamically expressed genes ordered across pseudo-time in fiber development stages. See also Additional file 4: Table S3. **d** The Quantitative Real-Time PCR (qRT-PCR) for the gene examples at different pseudo-time. Transcript levels were normalized to *Ubiquitin* (Ghir_D13G015430) and three technical replicates were analyzed. The scRNA-seq expression (left) and qRT-PCR (right) are displayed. **e** The differentially expressed genes (DEGs) between WT and *fl* in each scRNA-seq cell cluster. **f** The qRT-PCR validation of *GhMYB25-like* and *GhMYB25* showing WT enrichment in C2 and C3 DEGs, respectively. **g** Expression heatmap of scRNA-seq (left) and time-course RNA-seq (right) for 163 of WT enriched C2 DEGs. **h** The GO-terms of top 10 biological processes for the WT.C3 enriched genes in **g**

To investigate whether the five cell types represent a lineage, we used monocle3 to construct the single-cell trajectories for the OI cells collected from different developmental stages (− 2 to 2 DPA). We first obtained an unrooted trajectory graph for the five clusters C1–C5 for the combined data from WT and *fl* (Fig. 2a). To define the root of the trajectory, we employed published time-course bulk RNA-seq data to guide this process (Additional file 2: Table S1). We computed a correlation map on the UMAP embedding to measure the similarity of gene expression between single cells with bulk cells at a specific time point. Based on the correlation maps for − 3, − 1, 0, 1, and 3DPA data, we estimated the relative expression time for all single cells (Additional file 1: Fig. S3f-k and Additional file 4: Table S3). We found that the C1, C2, and C3 cells exhibit early, medium, and late temporal expression, while C4 and C5 cells do not exhibit significant changes during development. We thus determined that C1–C3 is the fiber cell lineage, in which the C1 cluster represents the cells at an early stage. We postulated that C4 and C5 are cortical cells or their derivatives, as cortical cells are the dominant population in the outer integument of ovules, and their gene expression patterns in WT and *fl* should be similar.

We further obtained the pseudo-time map (Fig. 2b) for the cells in clusters C1–C3 using monocle3 by setting the C1 cluster as the root node. We also identified the genes that change as cells progress along the pseudo-time and found that their temporal expression patterns matched with the bulk RNA-seq data (Fig. 2c). Along this developmental trajectory, we chose four genes with different pseudo-time expression patterns for validation using qRT-PCR for OI protoplast cells of both WT and *fl*. The results of the expression dynamics are consistent with the computational analysis (Fig. 2d), which further corroborate our inference on the development trajectory for C1–C3 (Fig. 2d).

Next, we investigated the key genes determining fiber cell development in WT. We reasoned that C2 represents the cell state closely upstream the C3 cluster, the fiber cells, and thus the genes enriched in C2 may play important roles in regulating fiber development. We identified differentially expressed genes (DEGs) between WT and *fl* at the single-cell level. In the putative epidermal cell lineage (C1–C3), C2 has the most upregulated DEGs (Fig. 2e and Additional file 3: Table S2). Strikingly, DEGs in C2 include *GhMML3/GhMYB25-like* (Ghir_D12G017660), the causal gene for fiber mutation, and its downstream-regulated gene *MYB25* (Ghir_D04G020730) [3], whose expressions from − 2 to 2 DPA were validated by qRT-PCR (Fig. 2f). We further investigated the relative expression of 163 WT enriched genes in C2 of the five cell clusters and in the time-course RNA-seq data. We found that a group of 38 genes with decreased expression in C3 show earlier expression starting at − 3 or − 1 DPA, such as *MYB25-like*, and another group of 116 genes with the highest expression in C3 show later expression starting at 0 or 1 DPA, such as *MYB25* (Fig. 2g), suggesting that C3 cluster might be originated from C2. Based on GO analysis, these C3 enriched genes are associated with trichome differentiation, plant epidermis development, and plant-type cell wall biogenesis, which are important processes associated with fiber development (Fig. 2h). Thus, C2 cells may serve as a transitionary state between C1 and C3 cells during the development of OI epidermal cells.

These findings strongly suggest that C1–C3 may represent the complete developmental trajectory for ovule OI epidermal cells, from the epidermal cell proliferation and differentiation to primary fiber cell elongation. Furthermore, the upregulated TFs in C2, including the causative gene *MYB25-like*, may act as pioneer activators in fiber primordia cells, inducing and determining cell differentiation fate to fiber cells, which is worth further experimental verification.

### Fiber cell-specific small peptides repress fiber cell growth

**Fig. 3:**
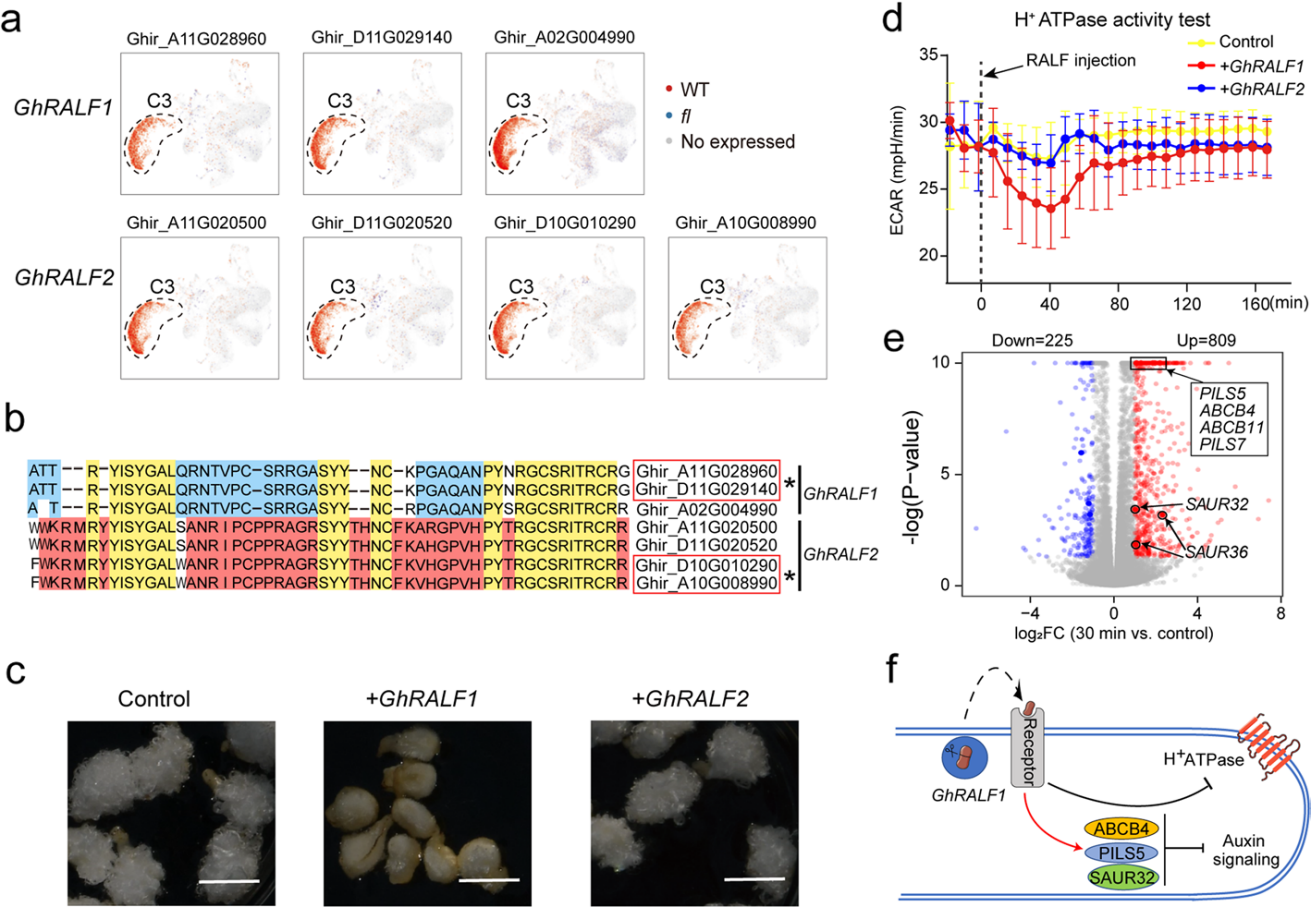
The small peptide GhRALF1s inhibits fiber cell growth. **a** UMAP projection showing the fiber cell-specific expression of *GhRALFs*. **b** The sequence alignment of matured GhRALFs. Conserved amino acids are labeled in green color, and specific amino acids of GhRALF1 and GhRALF2 are labeled in blue and red color respectively. **c** The fiber growth phenotypes of in vitro cultured ovules treated with GhRALF1 and GhRALF2. Scale bar, 5 mm. **d** The H + -ATPase activity test for 1 DPA ovules with GhRALF treatment measured by Seahorse XF24 Extracellular Flux analyzer, the *x*- and *y*-axis denote extracellular acidification rate (ECAR) and relative time to small peptide injection. Distilled water (pH 7.0) was injected as a control. The dashed line indicates the time point of peptide treatment. **e** Volcano plot for the GhRALF1 stimulated DEGs (FDR ≤ 5% and fold change ≥ 2). See also Additional file 5: Table S4. **f** The action mode for the inhibition role of GhRALF1 on fiber cells. The secreted peptide might be recognized by the receptor on PM to transduce inhibitory signals to proton pump and auxin signaling

Small secreted peptides have emerged as a vital taxon of cell-to-cell communication signals in plants. Interestingly, we found that 7 small secreted peptides of the RAPID ALKALINIZATION FACTOR (RALF) family [46] were highly expressed in C3 fiber cells (Fig. 3a and Additional file 3: Table S2). We analyzed the protein sequence similarities of their matured peptides and found that they can be classified into two subfamilies: GhRALF1 and GhRALF2 (Fig. 3b). We investigated their physiological roles on fiber cell growth by exogenously adding the synthetic peptides to the medium for in vitro culturing cotton ovules. We discovered that GhRALF1 can significantly in vitro inhibit fiber cell growth in a concentration-dependent manner (Additional file 1: Fig. S6a), whereas GhRALF2 has a limited effect on fiber growth at the same concentration, compared to the mock (Fig. 3c).

We first tested the roles of these RALFs on the proton pump activity of H^+^-ATPase for cotton ovules, as the peptides of the RALF family have been shown to cause apoplast rapid alkalization by inhibiting H^+^-ATPase activity on the plasma membrane (PM) in *Arabidopsis* [46]. We used two representative peptides from GhRALF1 and GhRALF2 for this proton pump activity assay. Under the injection with GhRALF1, we found that the ECAR (proton pump activity) dramatically drops, reaching the minimum around 40 min, and returns to normal levels within about 80 min post treatment, whereas GhRALF2 has no effect on the proton pump activity, showing almost the same trend as the control (Fig. 3d). We noted a transient decrease in ECAR after the control injection, which was due to the pH change caused by the addition of distilled water.

To further understand how the GhRALF1 functionally inhibits fiber growth in vitro, we performed RNA-seq to examine the gene expression changes for in vitro cultured ovules after a short-time (30 min) treatment with a synthetic GhRALF1 peptide. With two biological replicates, we identified 809 up- and 225 down-differentially expressed genes (DEGs) in the RALF-treated sample under the cutoffs of FDR ≤ 5% and fold change (FC) ≥ 2 (Fig. 3e and Additional file 5: Table S4). The upregulated DEGs are enriched in the auxin response pathway, including genes involved in auxin transport and signaling, according to gene function analysis with Metascape [47]. Specifically, the *PILS5* (Ghir_A02G019490) and *PILS7* (Ghir_D07G018780) that function in transporting auxin to the endoplasmic reticulum to reduce nuclear auxin signaling [48], *IAA13* (Ghir_A11G014220) that negatively regulates plant growth [49], and *SAUR32* (Ghir_A08G010800) and *SAUR36* (Ghir_A08G014400/D08G015270) that negatively regulate cell expansion [50], are all activated upon GhRALF1 treatment.

Together, these findings suggest that GhRALF1, small peptides highly expressed in fiber cells, can rapidly decrease the auxin signal response and repress the proton pump activity to inhibit fiber cell growth under in vitro conditions (Fig. 3f).

### The rhythmic growth regulation of fiber cells

**Fig. 4:**
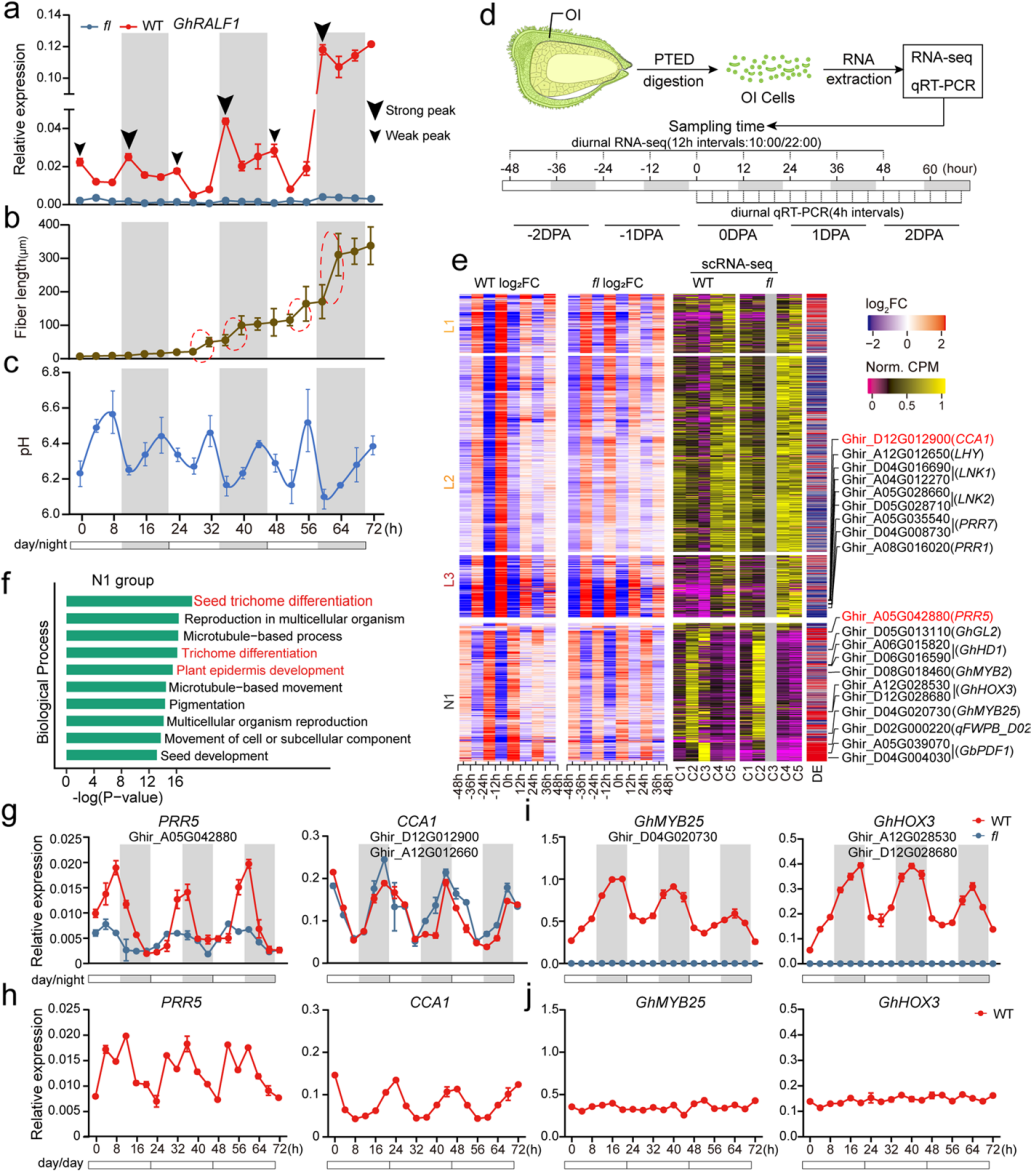
The rhythmic growth regulation of fiber cells. **a**–**c** The qRT-PCR of *GhRALF1* expression (**a**), fiber length dynamic (**b**), and apoplastic pH (**c**) for the fiber cells across 0–2 DPA. Night and day are shown in grey and white, respectively. Dotted circles highlight the fast elongation episodes of fiber growth. Big and small triangles indicate the strong and weak expression peaks. **d** The schematic illustration for diurnal RNA-seq for the OI protoplasts of ovules. **e** The diurnal rhythmic expression of the genes for the OI protoplast cells in WT and *fl* across − 2 to 2 DPA. The fold changes of gene expression heatmap (left) and significance of differential expression (DE) (middle) between neighboring time points, and gene expression level in each cluster for scRNA-seq data (right) were shown. The genes were split into four groups according to their expression pattern in the diurnal RNA-seq data. **f** The enriched top 10 GO terms of biological processes for N1 group in **e**. **g**–**j** The qRT-PCR validations for the expressions of core-clock genes (**g**, **h**) and fiber-associated genes (**i**,** j**) under normal day-night (day/night) and continuous light (day/day) conditions. Night and day are shown in grey and white, respectively

Due to the possible inhibitory roles of GhRALF1, the fiber cell growth would be hindered if the cells continue to express the peptide. Considering that the physiological effect is fast and temporary based on the above results (Fig. 3d,e), we hypothesized that *GhRALF1s* may exhibit temporal differential expression in fiber cells to avoid the putative long-term inhibition for primary fiber cell growth. To experimentally test this hypothesis, we used qRT-PCR to characterize the RNA expression pattern of 3 *GhRALF1*s for three consecutive days (0 to 2 DPA) at 4-h intervals. Notably, the transcriptional expressions of *GhRALF1s* in OI protoplasts of WT display a robust rhythmic fluctuation, with two expression peaks in 1 day: a strong peak at nightfall and a weak peak at daybreak, but this pattern is not observed in *fl* (Fig. 4a and Additional file 1: Fig. S6b-e). Furtherly, we found that *GhRALF1* keeps on day-night rhythmic fluctuation under continuous light (Additional file 1: Fig. S6f), indicating that its expression is controlled by the circadian clock.

Based on this circadian rhythmic expression pattern, we predicted that fiber cells may likewise show rhythmic growth. We thus monitored the fiber growth by measuring the length changes of fiber cells through serially sampling WT ovules at 4-h intervals for three consecutive days (0 to 2 DPA) for tissue sections (Additional file 1: Fig. S6g-h and see details in “Methods”). The statistical results demonstrated that early fiber growth (0 to 2 DPA) follows a typical daily cycle pattern, with two fast elongation episodes each day (Fig. 4b and Additional file 6: Table S5). Interestingly, the two fast elongation periods perfectly matched with the two times of apoplastic acidification (drops in pH value) of fiber cells on each day (Fig. 4c), suggesting that the rhythmic cell expansion of early fiber cells follows the acid growth theory as well. We noted that the *GhRALF1* transcription peaks appear to coincide with fiber cell elongation velocity peaks as well as apoplastic pH troughs, probably due to a temporal delay between transcription and physiological consequences as the peptides need time to be translated, secreted, and transduced. Another hypothesis is that GhRALF1 might in vivo promote fiber cell elongation, although a recent study also showed that RALF1 of Arabidopsis also inhibits cell growth in vivo [51]. Whether *GhRALF1* promotes or inhibits the growth of cotton fiber cells in vivo remains to be elucidated by further studies.

The robust rhythmic expression of *GhRALF1* inspired us to ask whether the genes associated with fiber development regulation show the oscillating expression patterns. To systematically investigate this problem, we conducted a diurnal transcriptomics analysis for the ovules OI protoplasts of WT and *fl* at − 2 to 2 DPA with two biological replicates, resulting in 36 RNA-seq samples (Fig. 4d,e and Additional file 1: Fig. S6i). As expected, 29,282 genes were identified to show day-night cyclic expression according to several strict criteria (See “Methods” and Additional file 7: Table S6). The genes could be classified into four groups (L1, L2, L3, N1) based on their day-night expression patterns (Fig. 4e).

Interestingly, the L3 group enriches plant clock genes including *PRR5/7/9*, *CCA1*, and *LNK1/2*. Most of these circadian oscillators display canonical day-night cyclic expression both in WT and *fl* (Fig. 4e and Additional file 1: Fig. S7a-b). We hypothesized that some core circadian oscillators might show expression differences between WT and *fl* due to fiber cell rhythmic growth. Expectedly, we did identify 25 differentially expressed clock genes with significance by comparing the day-night cyclic expression between WT and *fl* (Additional file 7: Table S6). For example, the RNA expression of *PRR5* in WT is higher than in *fl* (Additional file 1: Fig. S7b), and the qRT-PCR confirmed the significant amplitude difference of rhythmic expression across three consecutive days (0 to 2 DPA) between WT and *fl* (Fig. 4g, left). Because *PRRs* and *CCA1* have reciprocal repression to establish a feedback loop at the core of the circadian clock [52], the *CCA1* should show the opposite difference between WT and *fl*. The qRT-PCR for *CCA1* mRNA also verified this reciprocal repression (Fig. 4g, right). Furthermore, we examined the expression of these clock genes *PRR5* and *CCA1* under continuous light. We found that in the absence of physiological light clues, the two clock genes still show circadian rhythmic expression with the free-running period (FRP) (Fig. 4h).

We also noticed that the N1 group significantly enriches the terms of trichome differentiation (Additional file 1: Fig. S7a). Accordingly, the known cotton fiber-associated genes like *GhHD1*, *GhHOX3*, and *GhPDF1* are present in this group (Fig. 4e). We confirmed the rhythmic expression of the fiber-associated key TFs *GhHOX3* and *GhMYB25* by using qRT-PCR (Fig. 4i). Notably, no expression was detected for *GhHOX3* and *GhMYB25* in *fl*, showing that the rhythmic expressions of these key TFs are fiber cell-specific. However, under continuous light condition, the *GhHOX3* and *GhMYB25* changed to a constant expression pattern, losing the day-night rhythmic fluctuation (Fig. 4j). It shows that these key TFs for fiber cell development are light-responsive genes, whose expressions were strictly dependent on light.

In summary, we conclude that the primary fiber cell growth is a significant rhythmic (day-night) regulatory process, which is under the control of circadian clock genes and light-responsive genes, and the clock-controlled small peptides GhRALF1 might exert a rhythmic inhibiting role to regulate fiber growth during this process.

### TCPs rhythmically control translation and energy metabolism for fiber growth

We next attempted to understand the determinants at the chromatin level in regulating the circadian growth of fiber cells. We performed scATAC-seq assay for both WT and *fl* using the same samples as scRNA-seq. We probed 8992 and 13,615 cell nuclei, and detected a median of 13,490 and 10,540 unique genomic fragments per nucleus WT and *fl*, respectively (Additional file 2: Table S1). We identified 216,160 high confidence open chromatin peaks (Active chromatin region, ACRs) in total using MACS2 (Additional file 8: Table S7). The ACRs are widely distributed across the whole genome, with the highest enrichment at transcription start sites (Additional file 1: Fig. S8a-b), which conform with the typical features of ATAC-seq signals in plants [34]. To identify potentially distinct cell clusters based on scATAC-seq data, we constructed a single-cell binary matrix of chromatin accessibility based on all identified ACRs and performed dimension reduction analysis with t-SNE, PCA, and UMAP using SCALE and Seruat. However, no clear cell clustering was observed in either WT or *fl* by using any method (Additional file 1: Fig. S8c), indicating that the chromatin accessibility of the cells from the OI of the ovules largely remains similar in WT or *fl*, and the chromatin heterogeneity among the cells is insufficient for cell clustering.

Thus, we used the scATAC-seq data as bulk data to perform differential analysis between WT and *fl*, and identified 11,538 differentially accessible regions (DARs) under the cutoffs: fold change ≥ 2 and *p*-value < 0.01 (Additional file 1: Fig. S8d and Additional file 9: Table S8). At the gene level, there are 117 and 95 genes with differential chromatin accessibility (DAGs) that are enriched in WT and *fl*, respectively (Additional file 1: Fig. S8d). We found that there was no marker gene among those DAGs, and there were a small number of 23 marker genes harboring DAR(s), consistent with the chromatin heterogeneity being limited among cell clusters from the OI of the ovules. For most DAGs, their corresponding RNA expressions in different cell clusters positively correlate with chromatin accessibility (Additional file 1: Fig. S8e-f). The similar RNA expression pattern of top 25 WT or *fl* enriched genes in different cell clusters (C1–C5) further supports the homogeneity of chromatin accessibility for the different cell groups in cotton OI (Additional file 1: Fig. S8f). Two representative genes with the ATAC signals enriched in WT or *fl* are shown in Additional file 1: Fig. S8g.

**Fig. 5:**
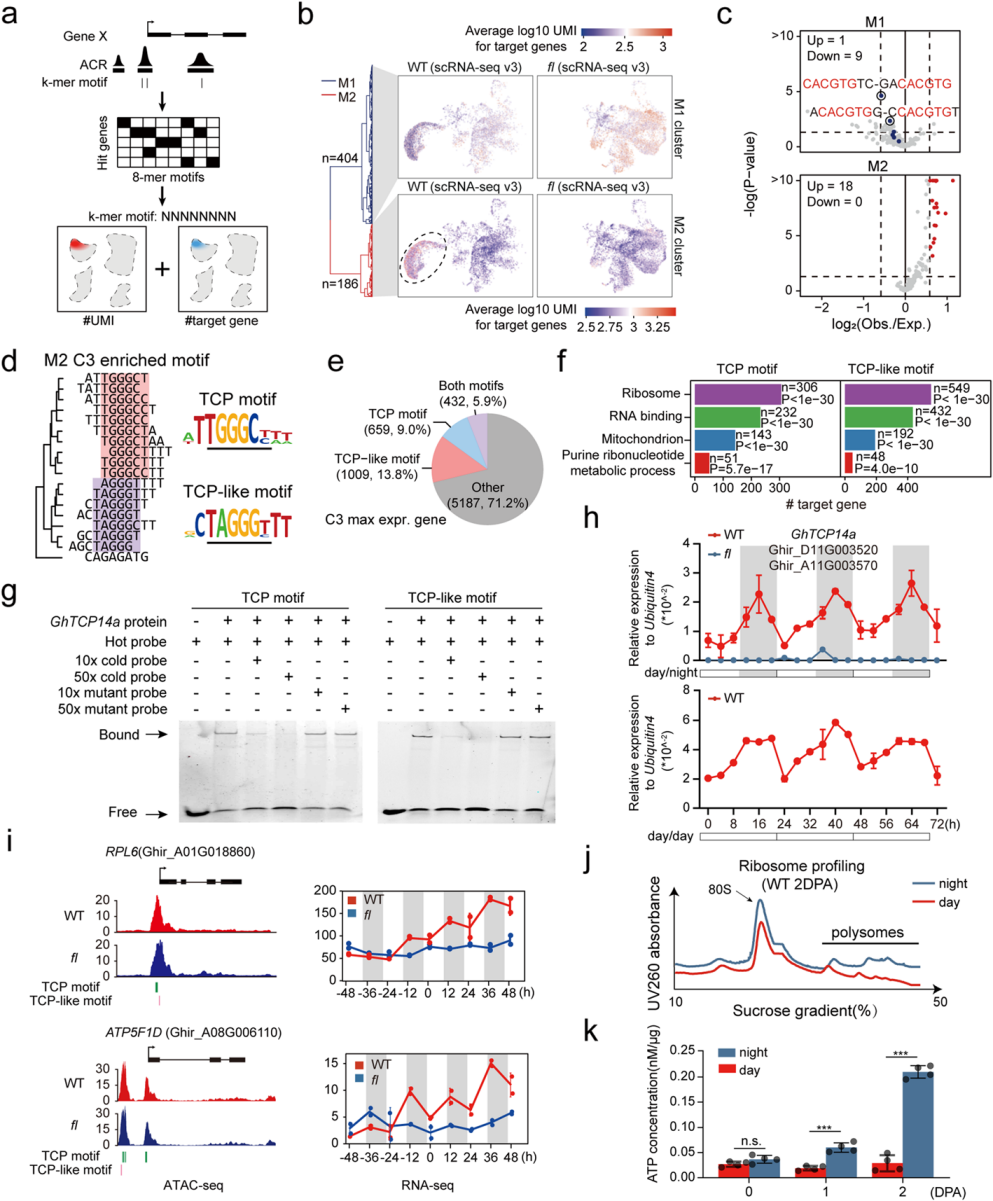
TCPs rhythmically control translation and energy metabolism during fiber growth. **a** Schematic illustration of the strategy for predicting fiber cell-specific *k*-mer motifs. First, count the *k*-mer motifs that appear in each ATAC-seq peaks and then use the positional relationship between ATAC-seq peaks and target genes to determine the relationship between *k*-mer motifs and targeting genes. Finally, count the overall impact of *k*-mer motifs on their targeting genes. **b** The clustering (left) and target gene expression distribution (right) for the two groups of fiber cell-specific 8-mer motifs. See also Additional file 10: Table S9. **c** The statistical significance for the fiber cell-specific *k*-mer motifs in M1 (left) and M2 (right). The *x*- and *y*-axis denote the observed/expected ratios (Obs./Exp.) of C3 maximum expressed genes and values of significance test (Fisher’s exact test), respectively. Dashed lines denote the thresholds (Obs./Exp. > 1.5 or < 0.67, *p*-value < 0.05). **d** The sequence alignment and logos for TCP and TCP-like motifs. **e** The pie chart shows the proportion of TCP and TCP-like motifs targeting genes in the genes with the highest expression in C3. **f** Enriched GO-terms for TCP (left) and TCP-like (right) motif targeting genes. **g** Gel shift assay showing GhTCP14a binds with both TCP and TCP-like motifs. **h** The qRT-PCR validations for the rhythmic expressions of *GhTCP14a* under normal day-night (day/night) and continuous light (day/day) conditions. **i** The rhythmic expressions (RNA-seq) of two gene examples involved in protein translation and energy supply. The ATAC-seq signals (left) and diurnal RNA-seq (right) were shown. The green and red lines below the tracks of ATAC-seq denote the locations of TCP and TCP-like motifs, respectively. The width of the lines reflects the number of motifs. **j** The ribosome profiling assays for the OI protoplasts of WT ovules at 2 DPA show the day-night cycle of protein translation in fiber cells. **k** The quantifications of ATP concentration for WT ovules at 0, 1, and 2 DPA show the day-night cycle of energy status in fiber cells. Asterisks denote a significant difference (unpaired Student’s *t* test, ***P < 0.001)

We further investigated potential regulators binding to the ACRs identified from ATAC-seq. We integrated the chromatin accessibility and scRNA-seq data and developed a pipeline to identify fiber cell-specific k-mer motifs in the ACRs in gene body or promoter region 1 kb upstream the TSS (Fig. 5a, Additional file 1: Fig. S9a-b, and see “Methods” for details). We identified 590 k-mers (*k* = 8) with strong potential in regulating specific genes and evaluated their relative distribution to the transcription start site (TSS) (Additional file 1: Fig. S9c-e). Based on the statistical tests in Additional file 1: Fig. S9c, we revealed that 317 k-mers are enriched around TSS, in which 247 k-mers show direction-specific occurrences (Additional file 1: Fig. S9d-e and Additional file 10: Table S9). Based on the metric measuring the expression correlation of *k*-mer’s target genes, we found that the 590 k-mers can be classified into two categories: M1 (*n* = 404) and M2 (*n* = 186), which represent two sets of *cis*-regulatory elements (CREs) that have inhibitory and activating roles on highest expressed genes in C3 fiber cells, respectively (Fig. 5b,c and Additional file 10: Table S9). Interestingly, the inhibitory M1 cluster contains several G-box motifs (Fig. 5c top and Additional file 11: Table S10), which were believed to be bound by clock gene PRRs to inhibit gene transcription in circadian rhythmicity regulation [53]. The result further supports the above conclusion that core circadian oscillators participate in the regulation of fiber cell development.

In addition, we found two groups of putative CREs in the activating cluster M2: TCP motif (TGGGCC/T) and TCP-like motif (TAGGGC/T) (Fig. 5c bottom, and Fig. 5d). Strikingly, about 1/3 of genes with the highest expression in C3 and about half of C3 marker genes have one of the two types of motifs or both (Fig. 5e, Additional file 1: Fig. S9f and Additional file 12: Table S11). The activating effect of TCP and TCP-like motifs is only observed in the C3 cluster (Additional file 1: Fig. S9g). Through further GO-term analysis, we found that the highly expressed genes in C3 potentially regulated by TCP and TCP-like motifs are significantly enriched in the biological processes of protein translation and mitochondrion (Fig. 5f).

We next analyzed all annotated *TCP* transcription factors and found that a specific branch (branch IV) of *TCP*s including *GhTCP7*, *GhTCP14*, and *GhTCP21* shows C3 fiber cell-specific expression (Additional file 1: Fig. S10a). Using gel shift assay, we verified that GhTCP14a (a *TCP14* member) can bind both TCP and TCP-like motifs in vitro (Fig. 5g). Interestingly, these genes in branch IV of the *TCP*s family show a strong diurnally oscillating expression pattern at 0–2 DPA in WT, but this rhythmic pattern is weak in *fl* based on the diurnal RNA-seq data (Additional file 1: Fig. S10b). We further used qRT-PCR to verify that the expression of *GhTCP14a* is controlled by the circadian clock, because it keeps on oscillating expression under either continuous light or normal light condition (Fig. 5h).

Notably, the target genes of these TCPs also exhibited significant rhythmic expression in WT, but weak or little in *fl* (Additional file 1: Fig. S10c). As shown in Fig. 5i, with strong ATAC signals at the TCP/TCP-like motifs in their promoters, the ribosome subunit-encoding genes *RPL9* and mitochondrial energy supply-associated genes *ATP5F1D* show significantly oscillating expressions in WT, the amplitude of which is much higher than that in *fl*. Consistent with the expression of the two representative genes, the protein translation and mitochondrial metabolism activities in WT are stronger than those in *fl* using ribosome profiling assays (Additional file 1: Fig. S10d) and oxygen consumption assays (Additional file 1: Fig. S10e), respectively. Furthermore, the activities of these two processes in WT ovules also exhibit day-night differences between day (AM 10:00) and night (PM 10:00) at 0, 1 DPA, and especially at 2 DPA, based on ribosome profiling (Fig. 5j and Additional file 1: Fig. S10f) and ATP measurement (Fig. 5k).

Together, the results revealed an important specific subfamily of TCP factors that circadianly regulate a large scale of fiber-associated genes to modulate the daily dynamics of mitochondria energy and protein translation in fiber cells.

## Discussion

In this study, we developed a novel PTED method to prepare high-quality protoplasts from the out integument cells of the ovules, and performed single-cell level analysis for gene expression and chromatin accessibility. The PTED can harvest enough protoplast cells of ovule OI from 8 to 10 cotton bolls at − 2 to 2 DPA in a short treatment time, eliminating the difficulty of in vitro manipulation to obtain the OI cells. For the first time, we captured the developmental trajectory of fiber lineage cells, discovered the diurnally oscillating fiber growth, and identified several new regulators including GhRALF1 and GhTCP14.

**Fig. 6:**
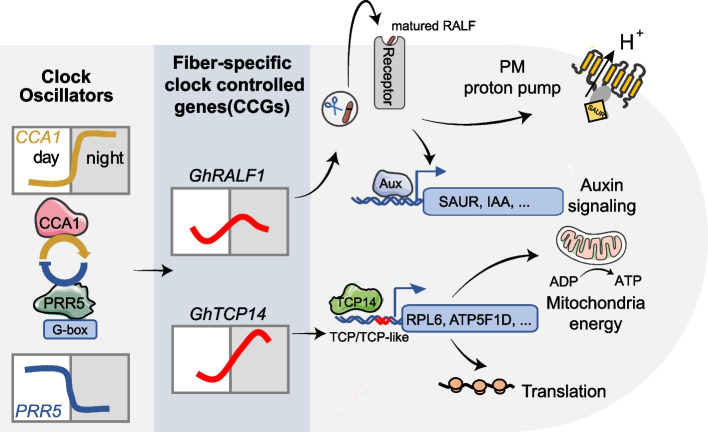
A model of circadian clock-orchestrated gene expression program in fiber cells. In fiber cells, the core circadian oscillators control the expression of the fiber-specific clock-controlled genes (CCGs), which further rhythmically regulates the metabolism dynamics for mitochondrial energy, protein translation, auxin signaling pathway, and plasma membrane proton pump

To our knowledge, our study conducted a high-resolution single-cell RNA analysis for the early growth of cotton fiber cells. We discovered that the fiber cells at early stages exhibit significant rhythmic characteristics, being a circadian clock-controlled rhythmic growth, based on multi-dimensional diurnal investigations including microscopy, physiology, and gene transcription. We sketched a regulatory network that is composed of the core-clock oscillators (*PRRs* and *CCA1*) and clock-controlled genes (CCGs), such as *GhRALF1* and *GhTCP14*, in the primary fiber cells (Fig. 6). The small peptide GhRALF1 may act as a rheostat during fiber cell growth, and their circadian fluctuating expression controls the rhythmic fiber cell growth through regulating proton pump and auxin signaling via *SAUR* and *IAA*. We proposed a novel paradigm that small peptides can function as circadian oscillators in regulating day-night cell growth in a concentration-dependent manner. In addition, we found that *GhTCP14* is another clock-controlled gene, which targets genes important for translation and mitochondria energy, such as *RPL6* and *ATP5F1D*. GhTCP14 was found to physically interact with HOX3 and MYB25 in a previous study [54], indicating that fiber-associated key TFs are linked with clock-controlled genes. Interestingly, the TCP14 homolog in Arabidopsis was found to directly interact with PRR5, a clock gene [55].

Unlike the centralized clock in mammals, the plant clock is proposed to operate as a decentralized hierarchy of locally coupled oscillators [56]. Tissues or even cells in the intact plant have been found to work as individual circadian clocks that oscillate with their own free-running period (FRP), which was revealed by single-cell bioluminescence imaging recently [57]. Our study discovered fiber cell circadian clock and its important molecular regulators. However, multiple key questions of the molecular mechanisms merit further investigation: (1) what is/are the entrainment signal(s) of fiber cell circadian rhythm; (2) how the circadian rhythm of fiber cells is coordinated with that of surrounding cells; (3) how do the core clock oscillators control fiber-specific CCGs, such as *GhRALF1* and *GhTCP14*, to modulate transcription, translation, physiology, and metabolism in fiber cells.

Notably, our study has shed light on exploring novel approaches for engineering circadian networks in cotton for improving fiber yield and quality. A variety of circadian clock genes have been linked to the agronomic traits of rice and other crops [58, 59]. Here, we have discovered that the circadian clock finetunes fiber growth, which is a key determinant of the most critical agronomic traits (length and quality) of cotton. Thus, reprogramming the circadian clock in fiber cells to modulate the link between fiber cell growth and circadian rhythm may obtain improved traits in fiber quality. According to previous studies, cotton cultivars grown in different geographic latitudes showed diversity in fiber lengths and other fiber quality traits [39, 40], as we reanalyzed in Additional file 1: Fig. S11a. We hypothesized that circadian clock changes caused by varied photoperiods in different geographic latitudes might contribute to the fiber diversity, in addition to potential climatic and geographic factors. Furthermore, we performed a preliminary experiment to study fiber growth under varying light conditions in greenhouse with constant temperature. Not unexpectedly, we observed significant difference of fiber lengths in two different photoperiods (normal and long day) during the blossoming and boll-forming stages. The fiber length under condition of long day cycle (20 h/4 h) increased by 11% than that under normal condition (16 h/8 h) (Additional file 1: Fig. S11b-c). We believe that further studies of the molecular mechanisms and systematic engineering of the fiber circadian rhythm would generate new cotton cultivars with improved fiber traits.

## Conclusions

By devising the PTED method for protoplast preparation and using scRNA-seq, scATAC-seq, and LCM-seq, we have successfully profiled the transcriptome of fiber cell and constructed the development trajectory of fiber cell lineage for the cotton ovules at primary development stage. Moreover, our results show that small peptide GhRALF1 and transcription factor GhTCP14 circadian rhythmically controls fiber cell growth possibly through oscillating the hormone signaling, extracellular pH, and the metabolism of mitochondria and protein translation. Altogether, we uncover for the first time that the primary growth of fiber cells is a highly regulated circadian rhythmic process under the control of a fiber-specific circadian gene expression program.

## Methods

### Plant materials and growth conditions

The upland cotton *G. hirsutum* cv. Xuzhou 142 (WT) and its mutant Xuzhou 142 fuzzless/lintless (*fl*) were cultivated in an automated greenhouse with mimicked natural conditions in setting 16 h light and 8 h dark for a day-night cycle, the temperature of 30 °C and humidity of 65%. For comparing fiber growth in different day-night cycles, the cotton plants at blossoming and boll-forming stages were transferred to the 20 h/4 h and 16 h/8 h cycles, respectively.

### In vitro* culture of the ovule and synthetic peptide treatment*

In vitro culture of ovule was performed referring to a previous study [60]. The peptide sequences of GhRALF1 and GhRALF2 after the canonical RRXL cleavage were synthesized (Dia-An Biotechnology, China). The peptide was added to the medium to a final concentration of 1 μM as treatment. The ovules were cultured at 28 °C in dark.

### Measurements of apoplastic pH and elongation rate for fiber cells

For detecting apoplastic pH and measuring the elongation rate of fiber cells, the apoplastic sap and tissue sections of ovules from 0 to 2 DPA were periodically prepared at 4-h intervals. The pH was measured with a microelectrode (METTLER TOLEDO, Germany). To avoid variations caused by phenotypic plasticity as much as possible, the fixed ovules were taken from the exact same position in row and column of the ovary locule. The tissue sections of ovules were made following our previous method [61] and stained with safranine O and fast green. The fiber lengths were imaged with a light microscope and measured with tools of CaseViewer (V2.4) (3DHISTECH, Hungary).

### *Tissue *in situ* hybridization*

The in vitro transcribed sense and antisense RNA probes were used in situ hybridization. The 14-μm sections were hybridized with the prepared probes and incubated overnight in an oven at 45 °C. The hybridization signals were produced with anti-digoxigenin-AP and NBT/BCIP kit (Roche, Germany) and acquired as images under a light microscope according to our previous method [61].

### Protoplast preparation

The equal number of ovules at five stages (− 2, − 1, 0, 1, and 2 DPA) was harvested from different cotton bolls followed by partial tissue enzymatic digestion (PTED). The ovules were digested with 1.5% cellulase R-10 (L0012, Yakult, Japan), 1.0% hemicellulose (H2125, Sigma-Aldrich, USA), 0.3% Macerozyme R-10 (L0021, Yakult, Japan) in 10 mM KCl, 2 mM MgCl_2_, 2 mM CaCl_2_, 0.1% BSA, 2 mM MES pH 5.7, and 600 mM mannitol for 1.5 h rotation (~ 50 rpm) at 28 °C. The ovules were then filtered with a 40-μm Falcon nylon mesh (Fisher scientific, USA) to remove undigested tissues and other large impurities. The filtrate was centrifuged for 10 min at 100 g. The protoplast pellet was retained and washed 3 times with 8% mannitol and finally resuspended in 500 μL 8% mannitol buffer. The number of protoplasts was counted using a hemocytometer. After trypan blue staining, only the protoplast sample with more than 85% viability was used.

### scRNA-seq and scATAC-seq library construction and sequencing

The scRNA-seq libraries were prepared using the Chromium Single Cell Gel Bead and 3’ Reagent Kit v2 and v3 (10 × Genomics, Pleasanton, CA). About 15,000–20,000 cells were loaded in the 10 × Genomics Chromium single-cell microfluidics (10 × Genomics, Pleasanton, CA) to generate single-cell gel beads in emulsion (GEMs). After size and quality check with Agilent 2100 Bioanalyzer, the libraries were sequenced on BGISEQ-500 (BGI, China) in PE100 mode.

For scATAC-seq, the fresh protoplasts were lysed with 10 mM Tris–HCL, pH 7.4, 10 mM NaCl, 3 mM MgCl_2_, 0.1% Tween-20, 1% BSA, 0.1% NP-40, 0.5% Dodecyl Maltoside (DDM), and filtered with a 40-μm nylon mesh (CORNING, USA). The nuclei were then spun down with 500 g for 3 min, resuspended, and purified using SH800S flow cytometer (Sony, Japan). For each sample, about 12,000 nuclei were used for Tn5 tagmentation and amplification following the previous protocol [62]. The MGISEQ-2000 (BGI, China) with PE50 mode was used for sequencing the libraries.

### Laser-capture microdissection and RNA sequencing (LCM-seq)

The laser-capture microdissection for fiber cells from cotton ovule sections is referred to a previous study [63]. The cotton ovules at 1 DPA were embedded in Tissue-Tek® OCT (Sakura, Tokyo, Japan), frozen immediately in liquid nitrogen, then cryosectioned. A 10-μm thickness was chosen to keep monolayer cells in *z*-axis as much as possible for each section, which minimized the contaminations from the cells in *z*-axis for thick sections. The fiber cells were individually harvested by laser-capture microdissection with cryomicrotome (Leica, Germany) and lysed in 0.2% Triton X-100. A total of 30 fiber cells were pooled as one replicate for sequencing. Three biological replicates were prepared. The low-input mRNA-Amplification Kit (N712, Vazyme, China) and TruePrep® DNA Library Prep Kit (TD503, Vazyme, China) were used in the RNA sequencing library construction. The libraries were sequenced on DNBseq-T7 (BGI, China) with PE100 mode.

### Protoplast RNA extraction and qRT-PCR validation

The protoplast cells from the out integument (OI) of ovules at different stages (− 2 DPA to 2 DPA) were harvested using the PTED method. RNA extraction was performed in following our previous method [61]. The total RNA was reverse transcribed by using EasyScript One-step gDNA Removal and cDNA Synthesis SuperMix (AE311-03, Transgen Biotech, China) with oligo-dT and random primers. The SYBR Green quantitative real-time PCR (qRT-PCR) was used for validating the differential RNA expression between WT and *fl* mutant. The *ubiquitin* gene (Ghir_D13G015430) was used as an internal control to perform 2^−ΔCT^ analysis.

### Measurement of the activity of H^+^-ATPase, oxygen consumption and ATP

By using Seahorse XF24 Extracellular Flux analyzer (Agilent, USA), the extracellular acidification rate (ECAR) and the oxygen consumption rate (OCR) of ovules from WT and *fl* mutant were examined. The method was adapted from a previous study [64, 65], which reflects the activity of the proton pump and mitochondrial respiration. The XF Calibrant Solution was added to hydrate the sensor at 28 °C overnight before the experiment. The synthesized peptide of GhRALF1/2 dissolved in distilled water (pH 7.0) was injected into the Seahorse XF Base Medium to the final concentration of 1 μM. Distilled water (pH 7.0) was used as a control. Three ovules per well and three replicates were performed for each treatment. The setting of measurement was mixing for 3 min, waiting for 2 min, and then measuring for 3 min, and the procedure runs for 23 cycles. The OCR and ECAR values were recorded by XF analysis software wave.

ATP concentration tests were measured with ATP assay kit (Beyotime, Shanghai, China). The ovules were lysed with 200μL lysis buffer and then centrifuged at 12,000* g* for 5 min at 4 °C. The supernatants were mixed with ATP working solution. The luminescence signals were detected with FlexStation 3 (Molecular Devices, USA). ATP concentrations were normalized by protein concentrations.

### Polysome profiling analysis

As described previously [66], the polysome extracts were resuspended in polysome extraction buffer and then loaded onto a 10–50% continuous sucrose gradient, which were freshly made in SWC1 ultracentrifuge tubes (344,059, Beckman, USA) using Gradient Master and Piston Gradient Fractionator and prepared by centrifugation at 35,000 rpm and 4 °C for 4 h. Each polysome fractionation was examined by determining the UV260 absorbance profile (model UV-6, ISCO, USA).

### Gel shift assay

The *GhTCP14a* was expressed with C-terminal GST tag in *E. coli* and purified with the GSTrap column. According to our previous method [61], the FAM-labeled and unlabeled DNA probes containing TCP and TCP-like motifs were used as hot and cold probes, respectively (Additional file 13: Table S12). The binding reactions were incubated at 25 °C for 60 min and electrophoresed on 6% native PAGE gels. Finally, fluorescence signals were detected using the Bio-Rad ChemiDoc MP Imaging System.

### RNA-seq data analysis

The adaptor in the raw sequencing reads were removed by cutadapt (v2.5) [67], and the cleaned reads were mapped to the *Gossypium hirsutum* genome [68] using STAR (v2.7.2b) [69] with following parameters (sjdb = 140, outFilterMultimapNmax = 5, outSAMmultNmax = 1, alignEndsType = Local, outFilterMatchNminOverLread = 0.4, outFilterScoreMinOverLread = 0.4, alignIntronMax = 100,000, alignMatesGapMax = 100,000). After removal of PCR duplicates using picard (v2.13.2, http://broadinstitute.github.io/picard/), gene expression quantifications in CPM (Count Per Million reads) were computed using the featureCounts program (v1.5.3) with default parameters [70]. Ovule tissues RNA-seq data from NCBI SRA database (Additional file 2: Table S1) were analyzed using StringTie (v2.0) [71] and TACO (v0.7.3) [72] to reconstruct genes, and those not appearing in HAU v1 annotation were merged as the gene annotation for all analyses in this study.

### Data processing of scRNA-seq

Cellranger (v3.1.0) was used to map and count reads with default parameters to generate the single-cell gene expression matrix. Empty cells were filtered out by the emptyDrops function in the DropletUtils package (v1.6.1) [73] with FDR cutoff = 0.01 and unique molecular identifier (UMI) cutoff = 1000 or 4000 for 10 × Genomics v2 or v3 libraries, respectively. To avoid cells being grouped into different clusters due to sequencing depth differences, two criteria were used to filter cells. First, the cells with a total UMI count larger than the median + 2 × MAD (median absolute deviation) value were removed. Second, for each cell, the numbers of detected and expressed genes were required to be in the range of 2.5–97.5% quantiles. The detected and expressed genes were defined as those with UMI counts in one cell ≥ 1 or ≥ 2, respectively.

### Removal of batch effects

High variation genes (HVGs) and modularity enriched genes (MEGs) were firstly identified for removing batch effects. HVGs were identified using the modelGeneVar function in scran package (v1.14.5) with FDR cutoff 0.05 [74]. The modularity scores were calculated by the clusterModularity function in scran for the 2990 genes (expressed in more than 100 cells and in less than 50% of total cells), and the 251 genes with modularity score tenfold larger than the background score were defined as MEGs.

The potential batch effects between all scRNA-seq data were eliminated using the correctExperiments function in the Batchelor package (v1.2.4) [75] based on the expression matrix of HVGs and MEGs with parameters: *k* = 25 and sigma = 0.3.

### Data dimensional reduction and cell clustering

The dimensionality of the batch effect-corrected expression matrix was reduced using the UMAP algorithm with the same setting as that in batch correction (umap.n_neighbors = 25, umap.min_dist = 0.3). In cell clustering, cells were first over clustered by monocle3 (v0.2.1) [76] with parameters: *k* = 25, num_iter = 5. The identified MEGs above were used to guide the merging of clusters with the following method.

For a MEG, we used the cells with UMI count larger than 1 to mark its location on UMAP. The gene distance of two MEGs was calculated based on the location proximity of those cells on UMAP. For MEGs $i$ and $j$, the gene distance with reference to $i$ is defined as ${Dist}_{i\to j}=\frac{\sum_{m in EC(i)}\sum_{n in KNN(k, m, EC(j))}dist(m,n)}{\left|EC\left(i\right)\right| \times k}$, where $\mathrm{dist}(m,n)$ represents the Euclidean distance of cell $m$ and cell $n$ on the UMAP projection; $\mathrm{EC}(i)$ represents the cells expressing gene $i$, and $|\mathrm{EC}\left(i\right)|$ represents the number of cells; $\mathrm{KNN}(k, m,\mathrm{ EC}(j))$ represents *k* nearest-neighbor cells in $\mathrm{EC}(j)$ closest to cell $m$ (*k* set as 5). The gene distance between $i$ and $j$ is defined as the average of ${Dist}_{i\to j}$ and ${Dist}_{j\to i}$. The distance matrix between all MEGs were clustered by the hclust function followed splitting into groups by cutree function in R. The groups of MEGs and their location on UMAP were used as a guide in merging over clustered cells into four clusters (C1, C2, C3, and C5), and the cells without MEGs were merged into cluster C4.

### Identification of marker genes and differentially expressed genes

The candidates of marker genes were predicted using findMarkers functions in scran package with the threshold (FDR < 1E − 9 and Foldchange > 2) for WT, *fl*, and merged data, separately. In considering the difference at expression level between transcription factors (TFs) and other genes, we used two different criteria to identify the marker genes. For TFs included in PlantTFDB [77], all of those candidate maker genes were considered marker genes. For a specific cluster, non-TF genes were considered as marker genes when satisfying the following three conditions: (1) The minimum expression ratio of this cluster in WT, *fl*, and merged data was greater than 0.01; (2) The minimum expression ratio was greater than 1.5 times the maximum expression ratio of other clusters in WT, *fl*, and merged data; (3) The maximum expression ratio in other clusters was less than 0.1.

The edgeR package (v3.28) [78] was used to identify differentially expressed genes (DEGs) between WT and *fl*, in different clusters with threshold FDR<0.05 and more than 2-fold change.

### Correlation analysis of scRNA-seq data with LCM-seq and fiber RNA-seq data

Genes with average CPM > 1 in all fiber samples were defined as fiber-expressed genes for LCM-seq and fiber bulk RNA-seq data. Their expression profiles were compared with the expression values of scRNA-seq clusters computed by the Average Expression function in Seurat package (v3.2.0) and the Spearman correlation coefficients were calculated. In comparing scRNA-seq and fiber sequencing data, we divided the UMAP of scRNA-seq data into different bins with a granularity of 0.5 × 0.5, and merged cells of each bin together to generate pseudo-bulk RNA-seq data. The Spearman correlation coefficients between fiber RNA-seq and pseudo-bulk RNA-seq data were computed for all bins separately.

#### Prediction of pseudo-time trajectory

We used monocle3 to predict the pseudo-time ordering of cells. To define the root node, we used a published time-course RNA-seq data sampled ovules at − 3DPA, − 1DPA, 0DPA, 1DPA, and 3DPA (Additional file 2: Table S1). Gene expression quantification in FPKM (fragments per kb per million reads) for these RNA-seq data was calculated by StringTie [71]. Ovule-expressed genes were identified requiring FPKM > 1 in at least one sample. Cells are divided into different areas on UMAP with a resolution of 0.2. In each area, the correlation between scRNA-seq and time-course RNA-seq data were calculated based on the expression level of those ovule-expressed genes (Additional file 1: Fig. S3g-k). The cell cluster C1 was identified as the starting point of early development. Since C4 and C5 were undistinguishable based on the time-course RNA-seq (Additional file 1: Fig. S3g), only those cells in C1, C2, and C3 clusters were used for pseudo-time trajectory analysis using C1 as the root node.

#### Gene function annotation and enrichment analysis

GO annotation for all genes was predicted by BLAST2GO, and the enriched GO terms were identified using topGO (v2.38) (http://www.bioconductor.org/packages/release/bioc/html/topGO.html). The information of transcriptional factors was downloaded from PlantTFDB database. Since the version of cotton genome sequence used in PlantTFDB (NUA-NBI) is different from the one we used (HAU), only the collinearity genes identified by MCScanX [79] with sequence identity larger than 90% between those two annotations were used for the gene ID conversion.

#### Identification of rhythmically expressed genes

The differential gene expression analyses between every pair of adjacent time points were firstly carried out with DESeq2 (v1.26) for WT and *fl* time-course RNA-seq data [80]. The expression changes were assigned as upregulated ( +) or downregulated ( −) based on the foldchange value. Potential rhythmically expressed genes (REGs) were called requiring that the expression levels periodically change (+ − or − +) in at least consecutive 72 h (6 pairs of changes) for WT or *fl* samples. In case the gene was rhythmically expressed in both WT and *fl* samples, the day-night change patterns were required to be the same. Significant REGs were defined as those REGs having significant expression differences (FDR < 0.05 and foldchange > 1.5) over consecutive 48 h.

#### Data processing of scATAC-seq

The sequencing reads of scATAC-seq were firstly mapped to the reference genome using cellranger-atac program (v1.2.0) with default parameters. The peaks were called on the merged reads from single cells using MACS2 (v2.1.1) with default parameters for WT and *fl* samples separately [81]. The peaks from WT and *fl* samples were merged together using BEDTools (v2.29.0) [82] as active chromatin regions (ACRs) and the UMI counts for all the ACRs were computed for individual cells. Based on the ACR count matrix, we used SCALE (v1.0.2) [83] and Seurat (v3.2.0) [84] with different settings for clustering the cells and found that the cells were not grouped into distinct clusters (Additional file 1: Fig. S8c). We thus treated scATAC-seq data as one cluster and used the AverageExpression and FindAllMarkers functions in the Seurat library to perform quantitative and differential expression analysis for the ACRs, respectively. The ACRs with more than twofold change between WT and *fl* samples were defined as differential active regions (DARs) and used in further analysis. The Homer (v4.10.4) [85] software was used to identify enriched motifs in DARs.

The peak counts within the gene body and the 1000 bp region upstream of the transcription start site were summed together as the ATAC-seq signals at the gene level. The differentially active genes (DAGs) were called in a similar manner with DARs.

#### Motif analysis for scATAC-seq ACRs

We developed a computational pipeline to identify cell-specific motifs in ACRs as briefly described in Fig. 5a and fully depicted in Additional file 1: Fig. S9a. We first defined the gene-associated ACRs as those in the gene body region or within 1 kb upstream of the TSS. We computed a 0/1 motif-gene matrix, in which “1” represents the presence of a specific *k*-mer (*k* = 8) motif in the ACR(s) of a specific gene, while “0” represents the absence. For each scRNA-seq cell, the gene number and total UMI of expressed 8-mer motif-associated genes were calculated. The former represents the effect of whether its targeted genes are activated, and the latter represents the effect of the 8-mer motif on gene expression level. For each 8-mer motif, the quantity information of expressed gene number and total UMI was then projected to the UMAP space, and motifs with Moran’s I scores of these two statistics both in the top 5% level were considered to be cell-specific motifs that can significantly regulate gene expression (Additional file 1: Fig. S9b). The hierarchical clustering of cell-specific motifs was calculated by using the ward.D2 algorithm based on the Euclidean distance of total scRNA-seq UMI counts of the targeted genes. According to clustering distance, the 590 motifs were clearly divided into two groups: M1 targeted with C3 to downregulate genes, and M2 targeted with C3 to upregulate genes (Fig. 5b,c). Finally, TCP and TCP-like motifs were identified by re-clustering of motifs in the M2 group (Fig. 5d).

To identify the direction-specific motifs, we first defined the relative direction (forward or reverse) of a specific *k*-mer motif to the corresponding gene and computed the relative distance to the transcript start site (TSS) as depicted in Additional file 1: Fig. S9c. For motifs enriched near the TSS, the median absolute deviation (MAD) of the relative distance distribution is small, and thus, the MAD values of the forward motif (MAD_forward_) and reverse motif (MAD_reverse_) can be used to evaluate whether the motif is directional. We computed the MAD of the relative distance to TSS for all cell-specific 8-mer motifs and randomly shuffled the same number of motifs in all ATAC-seq peaks for 100 times as a randomized control. For a cell-specific 8-mer motif, the MAD_forward_, MAD_reverse_, and MAD_random_ of random motifs were calculated separately. We defined a forward or reverse motif to be around TSS when the corresponding MAD is less than the average MAD_random_ minus two times the standard deviation of MAD_random_ controls. All *k*-mer motifs can be classified into 3 groups: “non-enriched around TSS” (none of the forward and reverse motif is around TSS), “directional motif” (one of the forward and reverse motif is around TSS), or “non-directional motif” (both the forward and reverse motifs are around TSS). We used the max(MAD_forward_, MAD_reverse_) and min(MAD_forward_, MAD_reverse_) of all motifs to visualize the relative distance distribution (Additional file 1: Fig. S9d-e). We defined a motif around TSS to be a regulator of an expressed gene (CPM > 1 in any scRNA-seq cluster) when the distance between the motif and TSS of the target gene is less than its MAD.

To determine the effect of TCP and TCP-like motifs on gene expression, we examined the relative expression of their target genes in the clusters C1–C5, and counted the proportion of genes with the highest expression values in each cell cluster. For each cell cluster, we calculated a *p*-value using the exact Fisher test on (*N*_motif targets_, *N*_motif targets in cluster_, *N*_expressed genes_, *N*_expressed genes in cluster_) to evaluate whether the motif regulates gene expression in a specific cluster. To further estimate the range of variations, we randomly selected the same number of genes to the motif target genes among all expressed genes for 10,000 times as controls, and used the standard deviation as the error bar.

## Supplementary Information

**Additional file1: Fig. S1.** The protoplasts prepared by PTED. (a) The flowers and bolls of cotton (Xuzhou 142 WT) at -3 to 2 DPA. (b) The viability of protoplast cells was assessed by trypan blue staining. (c) The median longitudinal sections for ovules before and after PTED digestions. OI, outer integument. II, inner integument. ES, embryo sac. Scale bar, 500 μm. **Fig. S2.** Clustering method for scRNA-seq data. (a) Distribution of modularity enrichment folds of 3000 expressed genes without batch effect removal. See the ‘Remove batch effects’ section in the Method. (b) Clustering and UMAP projection of 251 MEGs in (a). (c) The UMAP projection of the original clustering of scRNA-seq data. (d) The UMAP projection of the final clustering was obtained by filtering and merging the original clustering according to the MEGs distribution. (e) The expression correlation between v2 and v3 kits. (f) The gene number and UMI counts per cell for C1-C5 clusters. **Fig. S3.** Fiber cell identity analysis using scRNA-seq, LCM-seq, and fiber bulk RNA-seq data. (a) The examples of marker genes in each cell cluster. UMAP projections (left) and point plots (right) for each cell cluster are displayed. (b) The data reproducibility analysis of the three replicates of LCM-seq of fiber cells at 1 DPA. (c) The gene expression correlation analysis between the fiber cells at 1 DPA from LCM-seq and the cells in different clusters from scRNA-seq. (d) The data reproducibility analysis of the three replicates of bulk RNA-seq of isolated fiber cells at 5 DPA. (e) The gene expression correlation analysis between the fiber cells (5 DPA) from bulk RNA-seq and the cells in different clusters from scRNA-seq. (f) UMAP projection of estimated expression time for scRNA-seq cells. (g-k) UMAP projection of the correlation coefficient between scRNA-seq and time-course RNA-seq data. **Fig. S4.** The top 10 GO-terms for the marker genes in each scRNA-seq cluster. The left is for non-TFs (Transcription Factors) and the right is for TFs. **Fig. S5.** The fiber-associated genes from the literature and their expression from scRNA-seq in this study. (a) The references and experimental evidence from previous studies for the five marker genes in C3 cluster. (b) Single-cell UMAP profiles (top) and quantified gene expression values (CPM) in C1-C5 clusters (bottom) for the five marker genes in the C3 cluster. (c) The references and experimental evidence from previous studies for the six highly expressed genes in C3 cluster. (d) Single-cell UMAP profiles (top) and quantified gene expression values (CPM) in C1-C5 clusters (bottom) for the six highly expressed genes in C3 cluster. The cells in C3 cluster are marked with dotted line circles. The experiment methods in previous studies are annotated below the original figures, including RNA in situ hybridization, GUS reporter, and qRT-PCR. **Fig. S6.** The rhythmic expression of cotton fiber-associated genes. (a) The effects on fiber growth of in vitro cultured ovules under GhRALF1 and GhRALF2 treatments at four different concentrations (1 nM, 50 nM, 500 nM, and 1 μM). Scale bar, 5 mm. (b-e) The transcriptional expression of GhRALF1s (b-c) and GhRALF2s (d-e) revealed with RNA-seq and qRT-PCR. (f) The qRT-PCR validations for the expressions of GhRALF1 under continuous light conditions. (g-h) The statistical method for the fiber length dynamics. The sampling time-points across 0-2 DPA and the two observation regions on the ovules (chalazal and mid-region) were shown in (g). The representative sections for ovules at 4-hour intervals across 0-2 DPA were shown in (h). (i) Heatmap showing the Spearman correlation coefficient for time-course RNA-seq data. **Fig. S7.** The expression of core circadian oscillators during the growth of fibers. (a) The top 10 enriched GO-terms for L1-L3 and N1 groups. (b) ATAC-seq and diurnal RNA-seq for PRR5 and CCA1. ATAC-seq, diurnal RNA-seq, and qRT-PCR for PRR7 and PRR9. **Fig. S8.** Quality control and analysis of scATAC-seq data. (a) The distribution of scATAC-seq signal on the whole genome. (b) The distribution of scATAC-seq signal around transcription start site (TSS) and transcription termination site (TTS). (c) Four methods for dimensionality reduction projection of scATAC-seq of WT (top) and fl (bottom). (d) The number of differentially expressed ATAC-seq signals in DARs (left) and DAGs (right) level between WT and fl. See also Additional file 9: Table S8. (e) The correlation analysis for scRNA-seq and bulked scATAC-seq. (f) The top 25 DAGs in WT and fl respectively. The fold change of ATAC-seq signal (top) and single-cell RNA expression in each cell cluster (bottom) are shown. (g) Two representative gene examples in (f) shown with gene models, ATAC-seq signals, and scRNA-seq relative expressions. **Fig. S9.** The identification of TCP and TCP-like motifs and the expression analysis for their targeting genes. (a) Flowchart showing the computational pipeline for identifying cell-specific motifs. (b) The distribution of Morans’I score of targeting gene number and total UMI for each 8-mer motif. (c) Flowchart showing the pipeline for identifying direction-specific motifs. (d) The median absolute deviation of the relative position distribution between (reverse) cell-specific k-mer motifs and transcription start site. (e) Three examples of “non-enriched around TSS” (top), “directional-specific” (middle), and “non-directional” (bottom) cell-specific k-mer motifs in (d). (f) The Venn diagram depicts the fraction of TCP and TCP-like motifs targeting genes in the genes with the C3 marker genes. (g) The proportion of TCP (top) or TCP-like (bottom) targeting genes to the genes with the highest expression in C1-C5. The same number of genes as the TCP/TCP-like motif target genes were randomly selected 10,000 times as controls to count the highest expressed cell types. The mean value and standard deviation of the 10,000 replicates were used as the random value and the error bar for the random samples. The p-values of the Fisher test are denoted on the bars. **Fig. S10.** The rhythmic expression of TCP gene family and their targeting genes. (a) The TCP gene family in cotton was clustered into four branches according to protein sequence alignment. (b) The expression heatmap for the genes in TCP VI branch including TCP14, TCP7, and TCP21 based on diurnal RNA-seq data. (c) The gene expression heatmap of scRNA-seq (left) and RNA-seq (right) for the TCP targeting genes in fiber cells. The data in diurnal RNA-seq presents clear rhythmic expression in WT, but not in fl. The known fiber-associated genes, and the genes involved in protein translation and mitochondrial energy were highlighted. (d) The translational activity comparisons with ribosome profiling assays for WT and fl ovules at 0-2 DPA. (e) The activity comparisons of oxygen consumption for WT and fl ovules. The x- and y-axis denote the time and OCAR values, respectively. (f) The ribosome profiling assays for 0-1 DPA WT ovules at night (10 PM) and day (10 AM). **Fig. S11.** The effects of day-night conditions on fiber lengths. (a) The statistics of fiber length for the cotton grown at different sites in China. The locations were indicated on the map of China (bottom). Data were collated from the previous report. (b) The phenotypes of gross plants, bolls, and seed fibers for the cotton plants under different day-night conditions. (c) The statistics of fiber length under ND (16h/8h) and LD (20h/4h). The values are the means ± s.d., *n*=100. Asterisks indicate a significant difference (*** *p*-value < 0.001; unpaired Student’s t-test).

**Additional file 2:** **Table S1.** Statistics of all sequencing data used in this study.

**Additional file 3:** **Table S2.** Gene list and expression levels of cell marker genes, the expression levels of DEGs in scRNA-seq, and the highly expressed genes in C3.

**Additional file 4:** **Table S3.** Pseudo-time analysis of scRNA-seq in C1, C2 and C3 cell clusters.

**Additional file 5:** **Table S4.** Gene list of DEGs under RALF stimulation.

**Additional file 6:** **Table S5.** Statistics for the fiber length dynamics.

**Additional file 7:** **Table S6.** Gene list and expression levels of circadianly expressed genes.

**Additional file 8:** **Table S7.** List of active chromatin regions (ACRs).

**Additional file 9:** **Table S8.** List of DARs and DAGs for scATAC-seq data.

**Additional file 10:** **Table S9.** List of 8-mer motifs and their target genes.

**Additional file 11:** **Table S10.** List of G-box containing genes with WT enriched ATAC-seq peak.

**Additional file 12:** **Table S11.** List of TCP motif and TCP-like motif target genes with the highest C3 expression.

**Additional file 13:** **Table S12.** PCR primers used in this study.

**Additional file 14.** Review history.

## Data Availability

The raw sequencing data from this study are deposited in the NCBI SRA database (https://www.ncbi.nlm.nih.gov/sra/) under project PRJNA847210 [86]. The published ovule RNA-seq were downloaded from SRA database (SRR1695181 / SRR1695182 / SRR1695183 / SRR1695184 / SRR1695185 / SRR2917183 / SRR2917184 / SRR2917185 / SRR2917186 / SRR2917187 / SRR2917188 / SRR8079305 / SRR8079306 / SRR16475115 / SRR16475114 / SRR16475108) [87–102]. *Gossypium hirsutum* genome was downloaded from Cottongen database [103]. The transcriptional factor gene list was downloaded from PlantTFDB database [104]. The source codes for the analysis are available at https://github.com/zhouyulab/scfiber [105] with MIT license in Github and at https://doi.org/10.5281/zenodo.7633152 in Zenodo [106].
