# Supplementary material for: Cell-specific clock-controlled gene expression program regulates rhythmic fiber cell growth in cotton
Source: Genome Biol. 2023 Mar 14;24:49. doi: 10.1186/s13059-023-02886-0 (PMC10012527; doi:10.1186/s13059-023-02886-0)
Supplement: Supplementary file 1 — Additional file1: Fig. S1. The protoplasts prepared by PTED. (a) The flowers and bolls of cotton (Xuzhou 142 WT) at -3 to 2 DPA. (b) The viability of protoplast cells was assessed by trypan blue staining. (c) The median longitudinal sections for ovules before and after PTED digestions. OI, outer integument. II, inner integument. ES, embryo sac. Scale bar, 500 μm. Fig. S2. Clustering method for scRNA-seq data. (a) Distribution of modularity enrichment folds of 3000 expressed genes without batch effect removal. See the ‘Remove batch effects’ section in the Method. (b) Clustering and UMAP projection of 251 MEGs in (a). (c) The UMAP projection of the original clustering of scRNA-seq data. (d) The UMAP projection of the final clustering was obtained by filtering and merging the original clustering according to the MEGs distribution. (e) The expression correlation between v2 and v3 kits. (f) The gene number and UMI counts per cell for C1-C5 clusters. Fig. S3. Fiber cell identity analysis using scRNA-seq, LCM-seq, and fiber bulk RNA-seq data. (a) The examples of marker genes in each cell cluster. UMAP projections (left) and point plots (right) for each cell cluster are displayed. (b) The data reproducibility analysis of the three replicates of LCM-seq of fiber cells at 1 DPA. (c) The gene expression correlation analysis between the fiber cells at 1 DPA from LCM-seq and the cells in different clusters from scRNA-seq. (d) The data reproducibility analysis of the three replicates of bulk RNA-seq of isolated fiber cells at 5 DPA. (e) The gene expression correlation analysis between the fiber cells (5 DPA) from bulk RNA-seq and the cells in different clusters from scRNA-seq. (f) UMAP projection of estimated expression time for scRNA-seq cells. (g-k) UMAP projection of the correlation coefficient between scRNA-seq and time-course RNA-seq data. Fig. S4. The top 10 GO-terms for the marker genes in each scRNA-seq cluster. The left is for non-TFs (Transcription Factors) and the righ [file 13059_2023_2886_MOESM1_ESM.docx]

Fig. S1

**
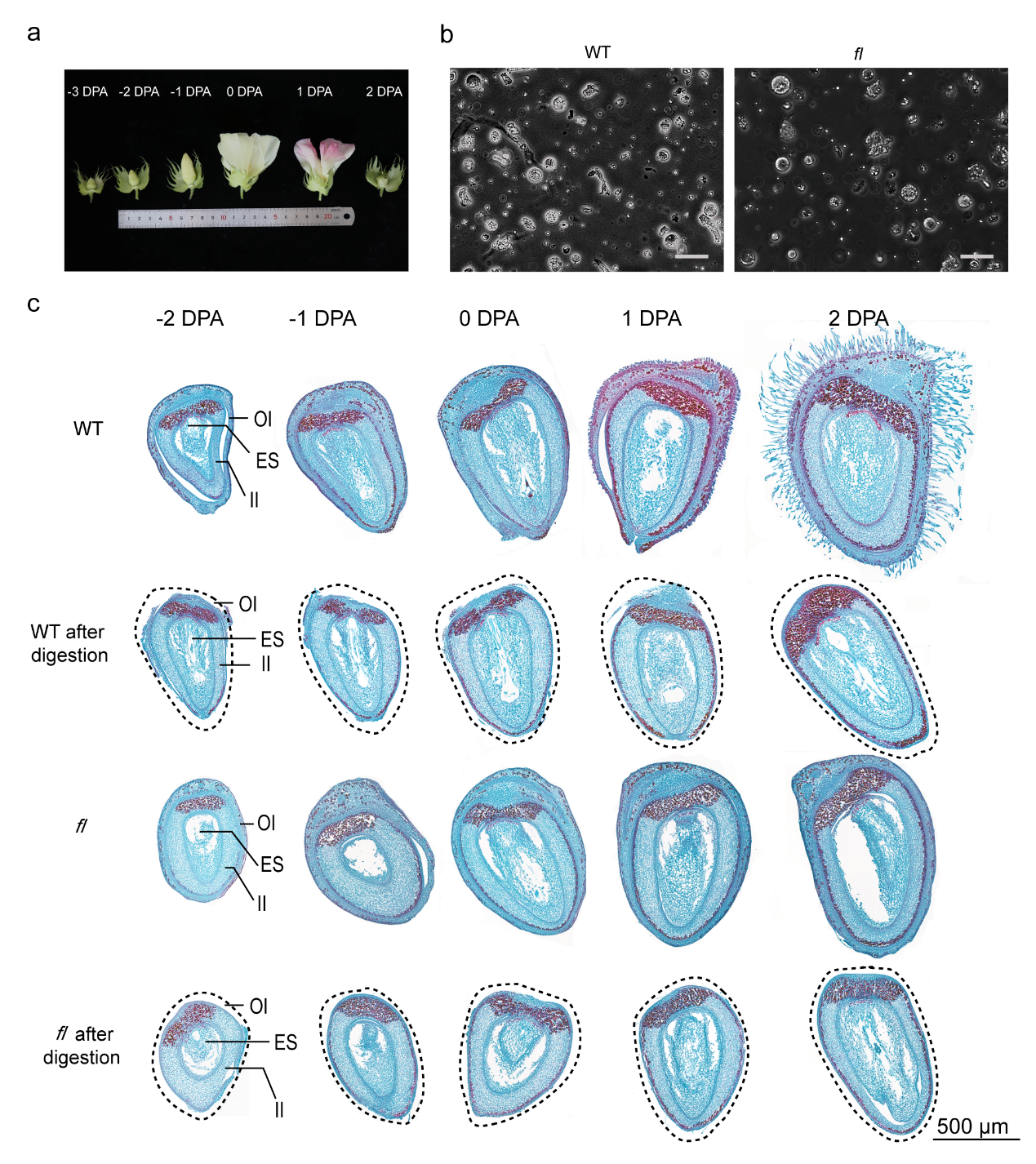
**

**Fig. S1** The protoplasts prepared by PTED. (**a**) The flowers and bolls of cotton (Xuzhou 142 WT) at -3 to 2 DPA. (**b**) The viability of protoplast cells was assessed by trypan blue staining. (**c**) The median longitudinal sections for ovules before and after PTED digestions. OI, outer integument. II, inner integument. ES, embryo sac. Scale bar, 500 μm.

Fig. S2


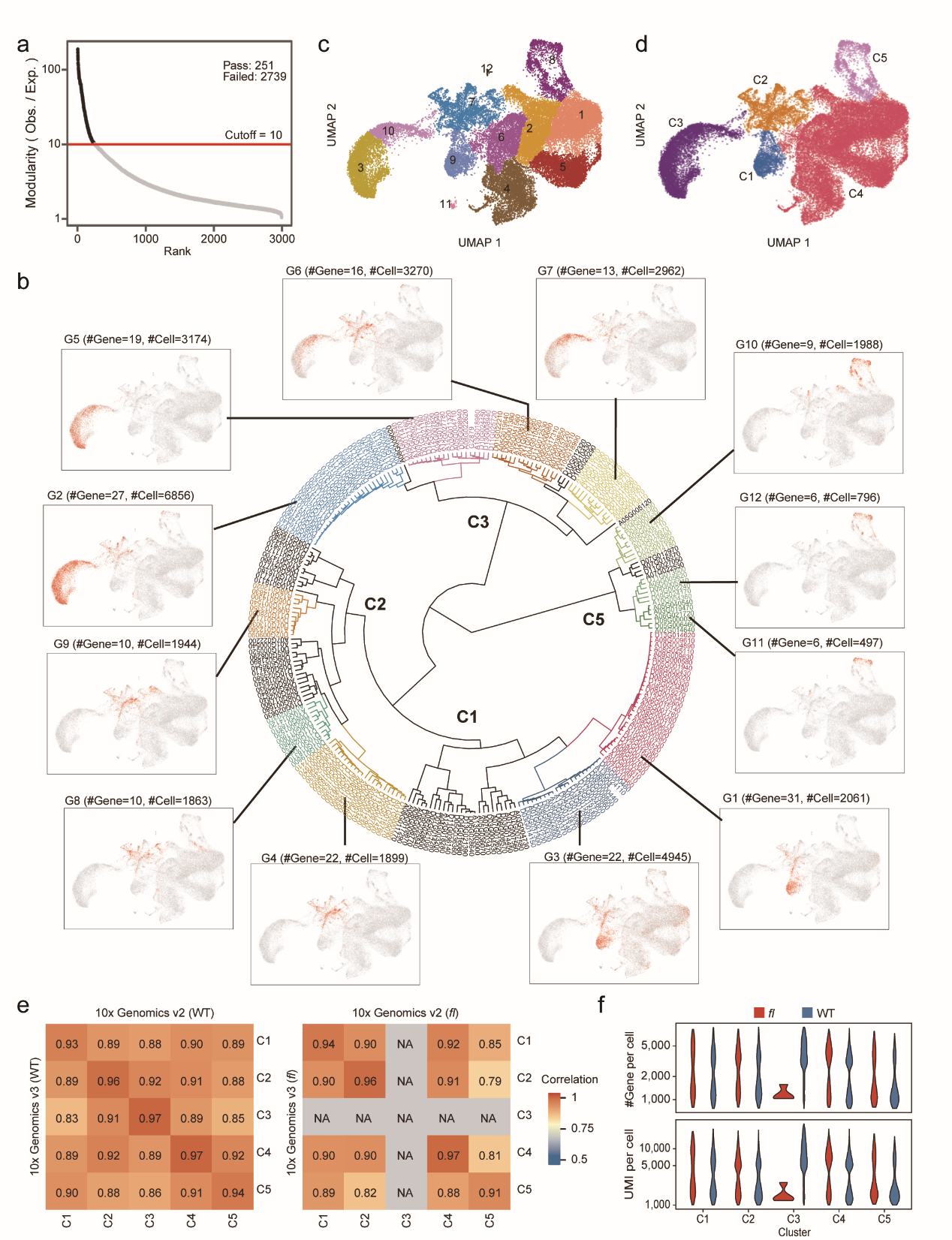


**Fig. S2** Clustering method for scRNA-seq data. (**a**) Distribution of modularity enrichment folds of 3000 expressed genes without batch effect removal. See the ‘Remove batch effects’ section in the Method. (**b**) Clustering and UMAP projection of 251 MEGs in (**a**). (**c**) The UMAP projection of the original clustering of scRNA-seq data. (**d**) The UMAP projection of the final clustering was obtained by filtering and merging the original clustering according to the MEGs distribution. (**e**) The expression correlation between v2 and v3 kits. (**f**) The gene number and UMI counts per cell for C1-C5 clusters.

Fig. S3


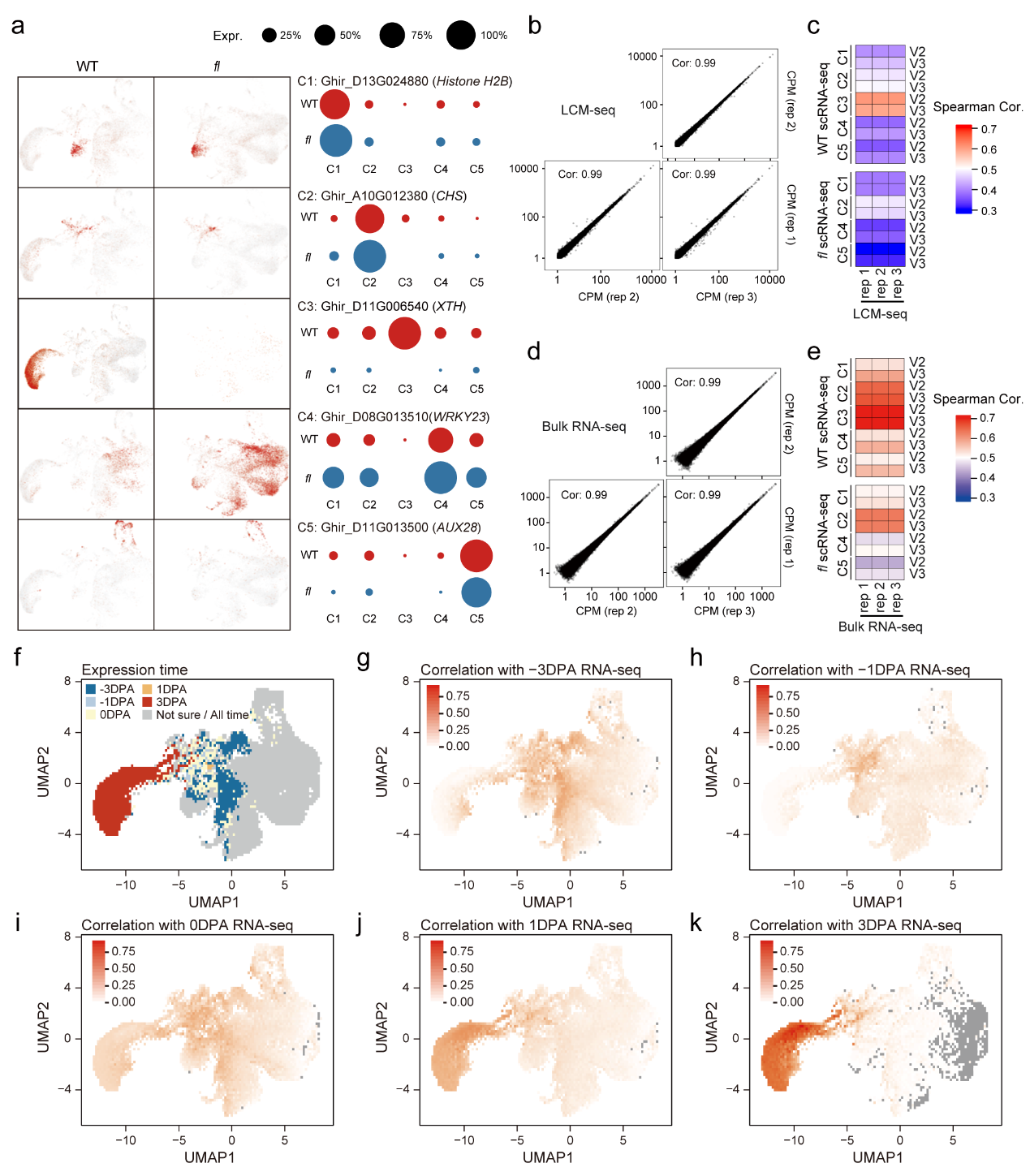


**Fig. S3** Fiber cell identity analysis using scRNA-seq, LCM-seq, and fiber bulk RNA-seq data. (**a**) The examples of marker genes in each cell cluster. UMAP projections (left) and point plots (right) for each cell cluster are displayed. (**b**) The data reproducibility analysis of the three replicates of LCM-seq of fiber cells at 1 DPA. (**c**) The gene expression correlation analysis between the fiber cells at 1 DPA from LCM-seq and the cells in different clusters from scRNA-seq. (**d**) The data reproducibility analysis of the three replicates of bulk RNA-seq of isolated fiber cells at 5 DPA. (**e**) The gene expression correlation analysis between the fiber cells (5 DPA) from bulk RNA-seq and the cells in different clusters from scRNA-seq. (**f**) UMAP projection of estimated expression time for scRNA-seq cells. (**g**-**k**) UMAP projection of the correlation coefficient between scRNA-seq and time-course RNA-seq data.

Fig. S4


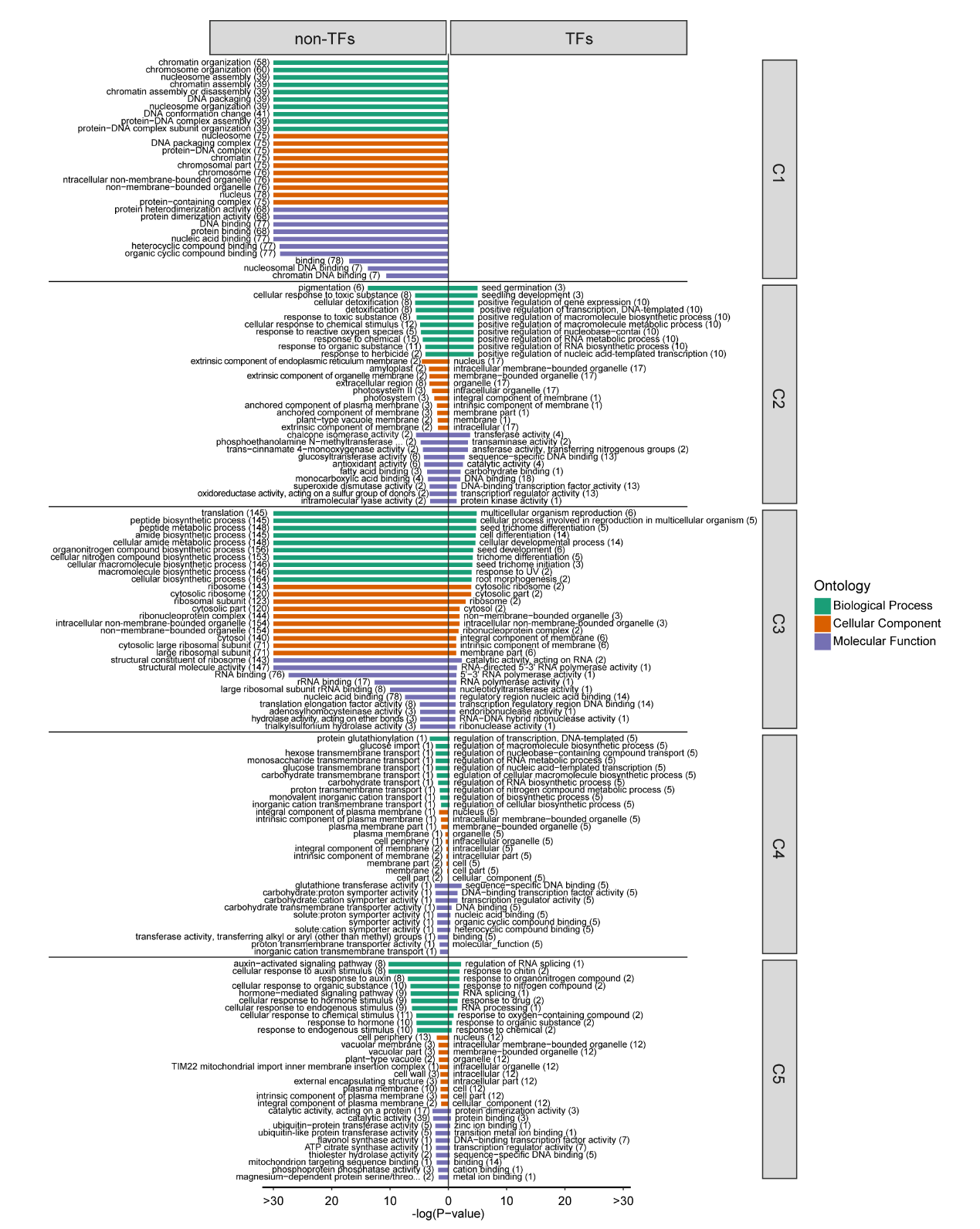


**Fig. S4** The top 10 GO-terms for the marker genes in each scRNA-seq cluster. The left is for non-TFs (Transcription Factors) and the right is for TFs.

Fig. S5


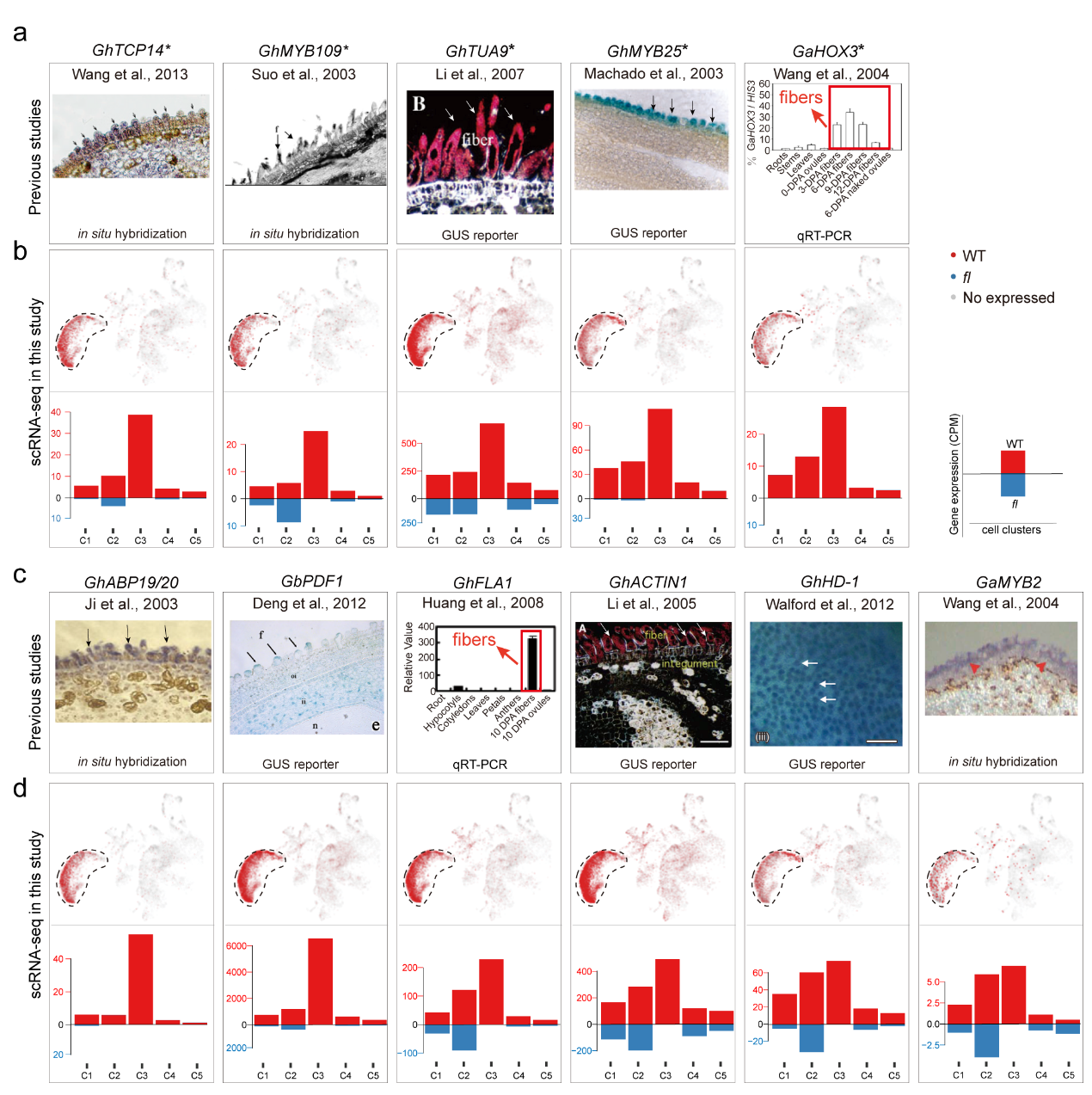


**Fig. S5** The fiber-associated genes from the literature and their expression from scRNA-seq in this study. (**a**) The references and experimental evidence from previous studies for the five marker genes in C3 cluster. (**b**) Single-cell UMAP profiles (top) and quantified gene expression values (CPM) in C1-C5 clusters (bottom) for the five marker genes in the C3 cluster. (**c**) The references and experimental evidence from previous studies for the six highly expressed genes in C3 cluster. (**d**) Single-cell UMAP profiles (top) and quantified gene expression values (CPM) in C1-C5 clusters (bottom) for the six highly expressed genes in C3 cluster. The cells in C3 cluster are marked with dotted line circles. The experiment methods in previous studies are annotated below the original figures, including RNA in situ hybridization, GUS reporter, and qRT-PCR.

Fig. S6


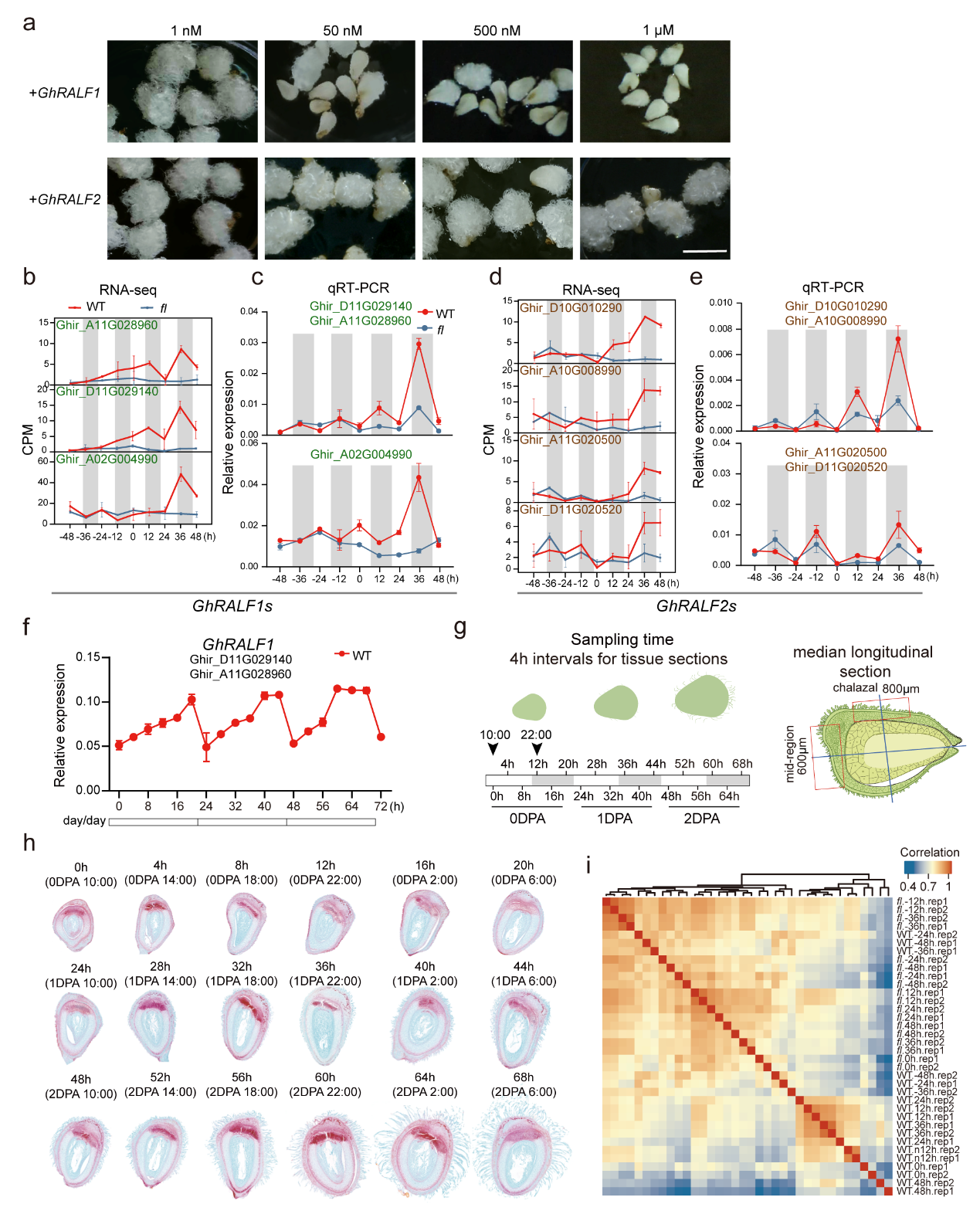


**Fig. S6** The rhythmic expression of cotton fiber-associated genes. (**a**) The effects on fiber growth of in vitro cultured ovules under GhRALF1 and GhRALF2 treatments at four different concentrations (1 nM, 50 nM, 500 nM, and 1 μM). Scale bar, 5 mm. (**b**-**e**) The transcriptional expression of *GhRALF1s* (**b**-**c**) and *GhRALF2s* (**d**-**e**) revealed with RNA-seq and qRT-PCR. (**f**) The qRT-PCR validations for the expressions of *GhRALF1* under continuous light conditions. (**g**-**h**) The statistical method for the fiber length dynamics. The sampling time-points across 0-2 DPA and the two observation regions on the ovules (chalazal and mid-region) were shown in (**g**). The representative sections for ovules at 4-hour intervals across 0-2 DPA were shown in (**h**). (**i**) Heatmap showing the Spearman correlation coefficient for time-course RNA-seq data.

Fig. S7

**
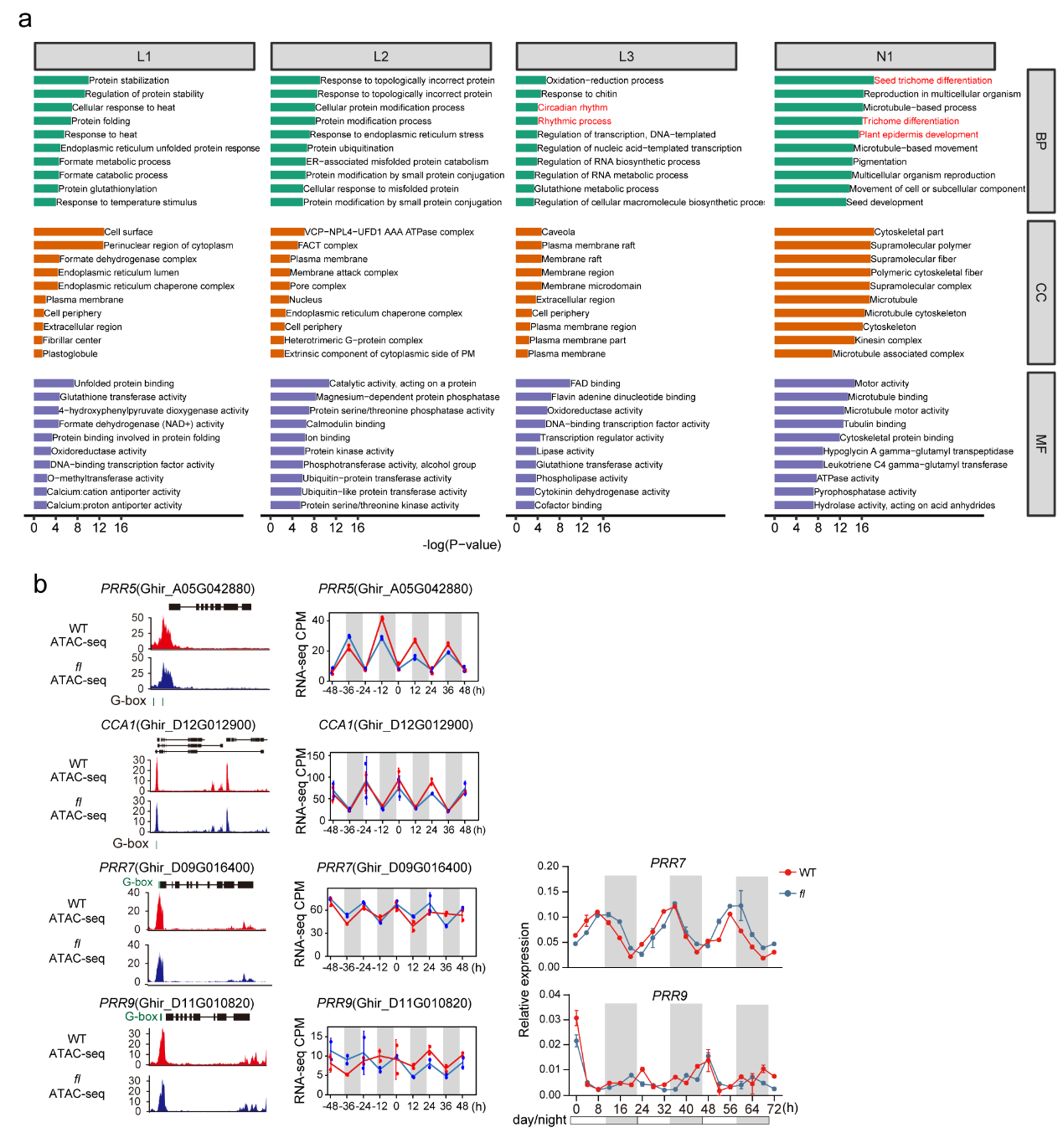
**

**Fig. S7** The expression of core circadian oscillators during the growth of fibers. (**a**) The top 10 enriched GO-terms for L1-L3 and N1 groups. (**b**) ATAC-seq and diurnal RNA-seq for *PRR5* and *CCA1*. ATAC-seq, diurnal RNA-seq, and qRT-PCR for *PRR7* and *PRR9*.

Fig. S8

**
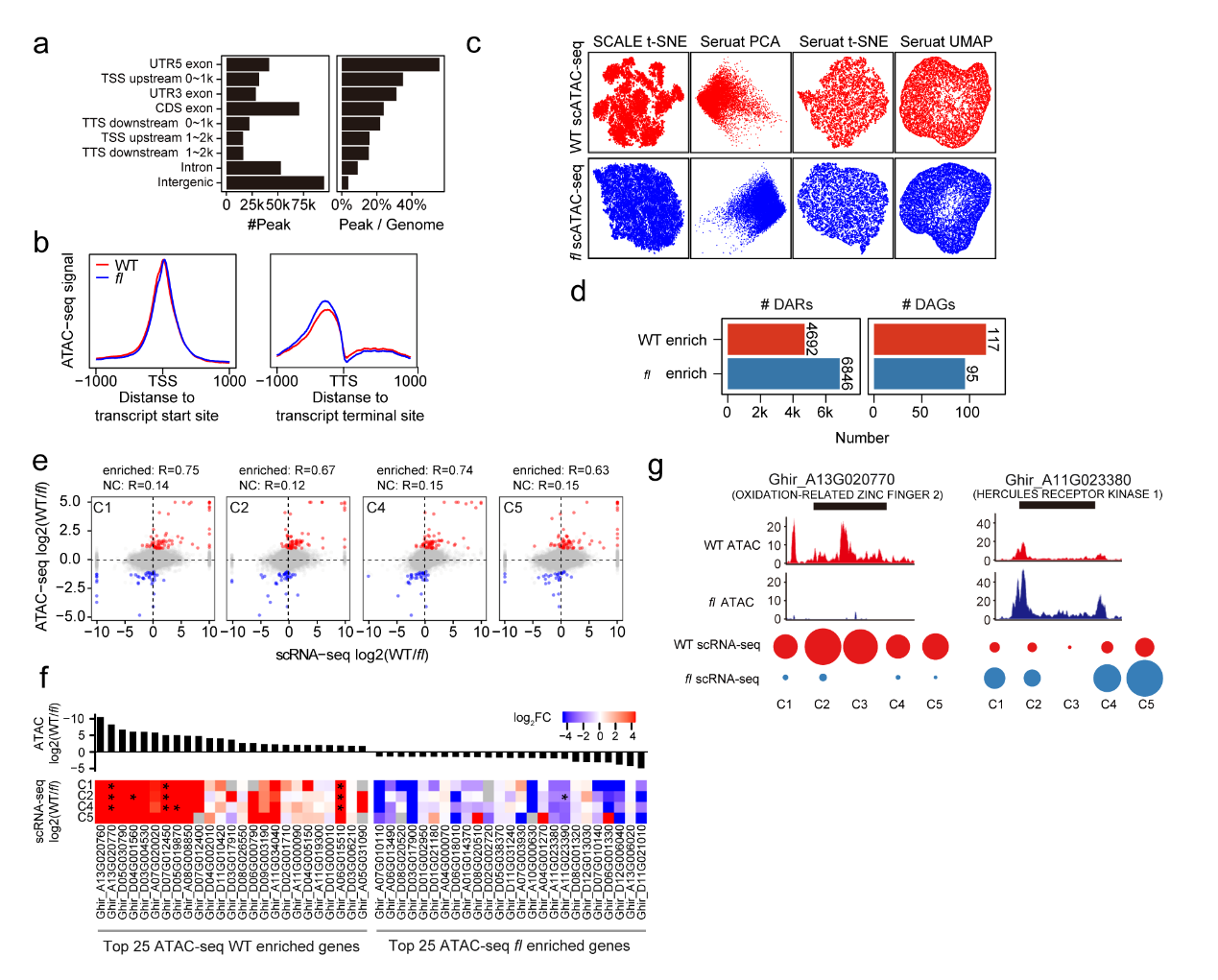
**

**Fig. S8** Quality control and analysis of scATAC-seq data. (**a**) The distribution of scATAC-seq signal on the whole genome. (**b**) The distribution of scATAC-seq signal around transcription start site (TSS) and transcription termination site (TTS). (**c**) Four methods for dimensionality reduction projection of scATAC-seq of WT (top) and *fl* (bottom). (**d**) The number of diﬀerentially expressed ATAC-seq signals in DARs (left) and DAGs (right) level between WT and *fl*. See also Additional file 9: Table S8. (**e**) The correlation analysis for scRNA-seq and bulked scATAC-seq. (**f**) The top 25 DAGs in WT and *fl* respectively. The fold change of ATAC-seq signal (top) and single-cell RNA expression in each cell cluster (bottom) are shown. (**g**) Two representative gene examples in (f) shown with gene models, ATAC-seq signals, and scRNA-seq relative expressions.

Fig. S9

**
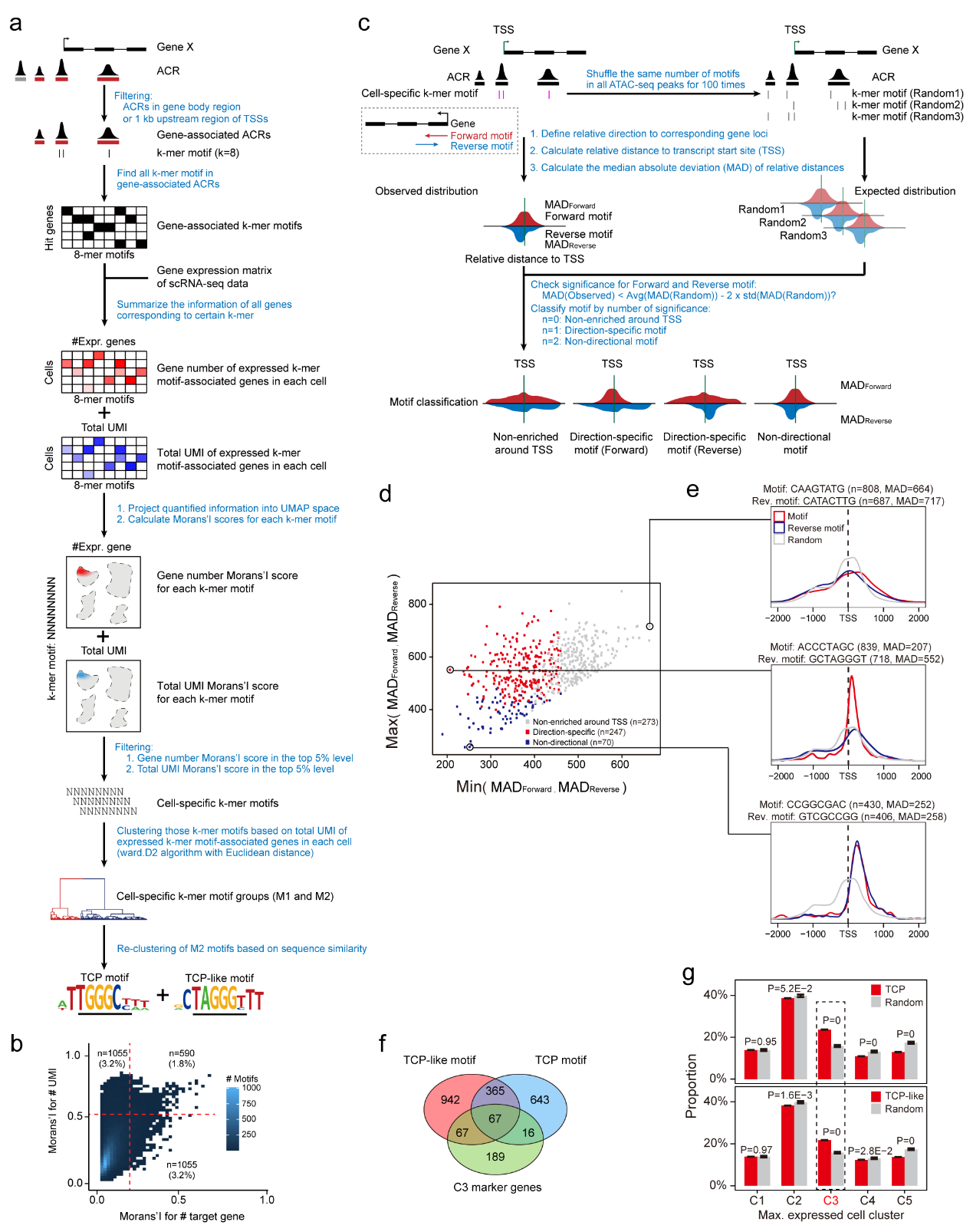
**

**Fig. S9** The identification of TCP and TCP-like motifs and the expression analysis for their targeting genes. (**a**) Flowchart showing the computational pipeline for identifying cell-specific motifs. (**b**) The distribution of Morans’I score of targeting gene number and total UMI for each 8-mer motif. (**c**) Flowchart showing the pipeline for identifying direction-specific motifs. (**d**) The median absolute deviation of the relative position distribution between (reverse) cell-specific k-mer motifs and transcription start site. (**e**) Three examples of “non-enriched around TSS” (top), “directional-specific” (middle), and “non-directional” (bottom) cell-specific k-mer motifs in (**d**). (**f**) The Venn diagram depicts the fraction of TCP and TCP-like motifs targeting genes in the genes with the C3 marker genes. (**g**) The proportion of TCP (top) or TCP-like (bottom) targeting genes to the genes with the highest expression in C1-C5. The same number of genes as the TCP/TCP-like motif target genes were randomly selected 10,000 times as controls to count the highest expressed cell types. The mean value and standard deviation of the 10,000 replicates were used as the random value and the error bar for the random samples. The *p*-values of the Fisher test are denoted on the bars.

Fig. S10

**
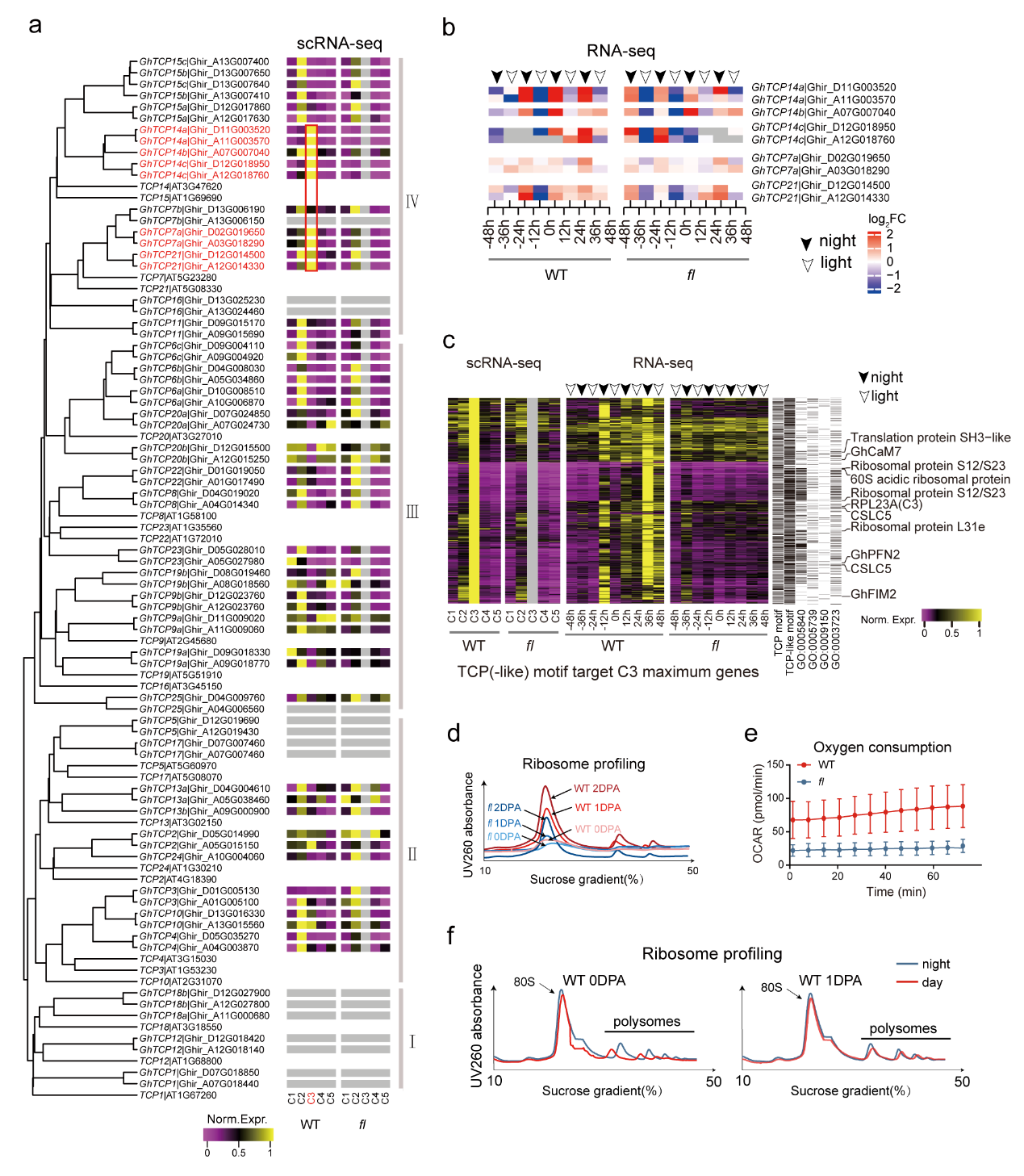
**

**Fig. S10** The rhythmic expression of TCP gene family and their targeting genes. (**a**) The TCP gene family in cotton was clustered into four branches according to protein sequence alignment. (**b**) The expression heatmap for the genes in TCP VI branch including *TCP14*, *TCP7*, and *TCP21* based on diurnal RNA-seq data. (**c**) The gene expression heatmap of scRNA-seq (left) and RNA-seq (right) for the TCP targeting genes in fiber cells. The data in diurnal RNA-seq presents clear rhythmic expression in WT, but not in *fl*. The known fiber-associated genes, and the genes involved in protein translation and mitochondrial energy were highlighted. (**d**) The translational activity comparisons with ribosome profiling assays for WT and *fl* ovules at 0-2 DPA. (**e**) The activity comparisons of oxygen consumption for WT and *fl* ovules. The x- and y-axis denote the time and OCAR values, respectively. (**f**) The ribosome profiling assays for 0-1 DPA WT ovules at night (10 PM) and day (10 AM).

Fig. S11

**
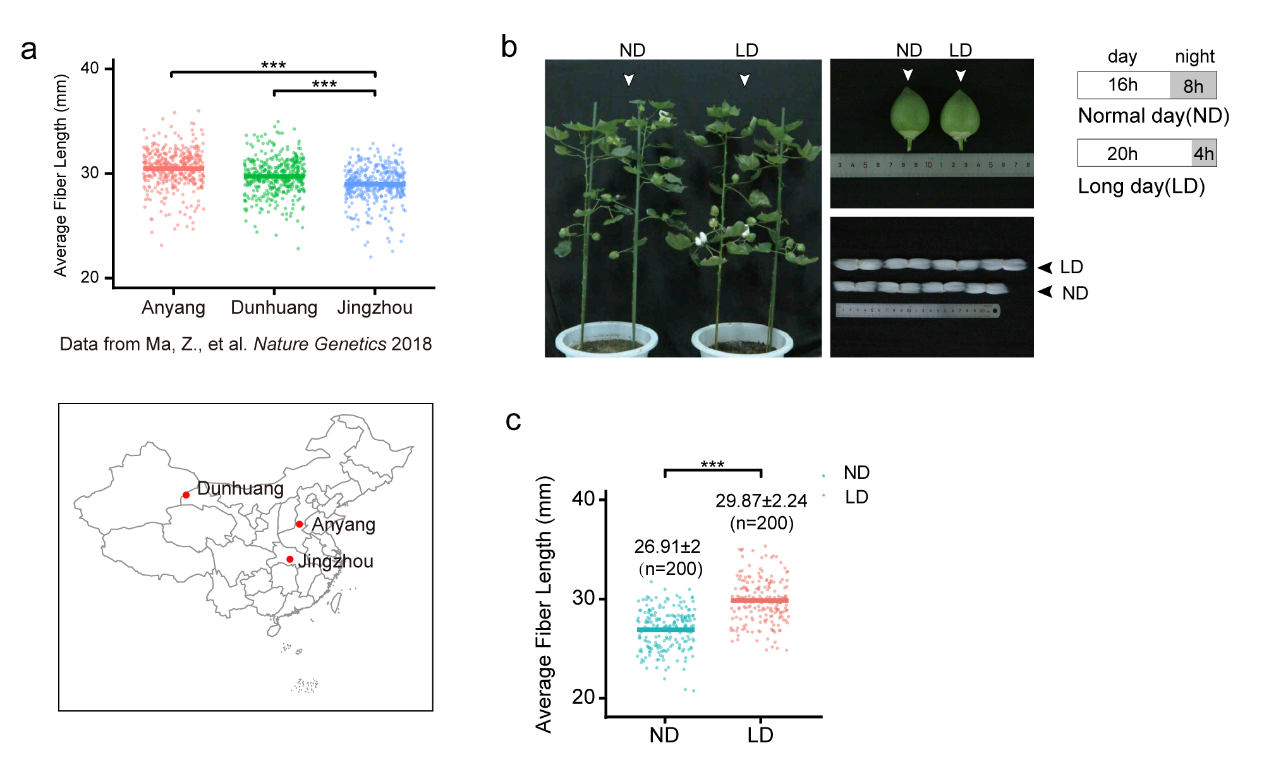
**

**Fig. S11** The effects of day-night conditions on fiber lengths. (**a**) The statistics of fiber length for the cotton grown at different sites in China. The locations were indicated on the map of China (bottom). Data were collated from the previous report. (**b**) The phenotypes of gross plants, bolls, and seed fibers for the cotton plants under different day-night conditions. (**c**) The statistics of fiber length under ND (16h/8h) and LD (20h/4h). The values are the means ± s.d., n=100. Asterisks indicate a significant difference (*** *p*-value < 0.001; unpaired Student’s *t-*test).
