# Supplementary material for: Cell-specific clock-controlled gene expression program regulates rhythmic fiber cell growth in cotton
Source: Genome Biol. 2023 Mar 14;24:49. doi: 10.1186/s13059-023-02886-0 (PMC10012527; doi:10.1186/s13059-023-02886-0)
Supplement: Supplementary file 14 — Additional file 14. Review history. [file 13059_2023_2886_MOESM14_ESM.docx]

Review History

**First round of review**

**Reviewer 1**

**Were you able to assess all statistics in the manuscript, including the appropriateness of statistical tests used?**

No, I do not feel adequately qualified to assess the statistics.

**Comments to author:**

Wang et al performed single cell RNA sequencing using the cells of Gossypium hirsutum outer integument (OI) of ovules from wildtype (WT) and fuzzless/lintless (fl) cotton. The authors annotated the cell type of C3 cell cluster as cotton fiber cells based on the following arguments: (1) Comparing the ovules in WT, the fl ovules exhibit deficiency on fiber initiation. Through the comparison of cell clusters between WT and fl, C3 cell cluster is absence in fl. (2) Some marker genes enriched with GO functional terms as trichome differentiation and initiation. (3) Some genes in C3 cell cluster are associated with cotton fiber. (4) Genes specifically expressed in cotton fiber cells.

Here are my comments toward these four arguments:

For argument (1), the absence of C3 cell cluster in fl could be caused by different reasons. For example, fiber initiation may require a type of "supporting cells", and the failure of initiation may be caused by the absence of "supporting cells" instead of the absence of fiber cells. In addition, an obvious decrease of cells in C5 cluster also appeared in fl, which can also be annotated as fiber cotton cells or the unknown "supporting cells".

For argument (2), the authors only selected the GO terms in C3 cluster that they favored for cell type annotation. Many other GO terms, instead, represent the majority and were not used for cell type annotation, such as organism reproduction, response to UV, root morphogenesis for transcription factors, and peptide biosynthetic process, cellular nitrogen compound biosynthetic process and amide biosynthetic process for non-transcription factors.

For argument (3), genes associated with cotton fiber does not mean that these genes can serve as marker genes for cell type annotation. Take MYB25 as an example, the association of MYB25 and fiber cotton is illustrated by the regulation of MYB25 toward fiber development. However, numerous studies have shown that regulations of certain developmental processes can be exerted by non-cell-autonomous pathways. Namely, it is not necessary for a gene to be specifically expressed in cotton fiber cells to regulate their development.

For argument (4), the authors seemed to misinterpret the conclusions from previous studies. Take a reference provided in the manuscript as an example, in Wang et al 2013, the following sentence was extracted: "Because it is technically unfeasible to separate the fiber initials from the ovules at early developmental stages, in situ hybridization was conducted to examine the expression of GhTCP14 in 0-DPA ovules when cotton fibers initiate from the ovule epidermal cells. GhTCP14 transcripts were also abundant in the fiber initials (Fig. 7B)." GhTCP14 was reported as an abundantly expressed gene not as a specifically expressed gene in cotton fiber, but this reference was used as a supporting evidence in this manuscript for specific gene expression of GhTCP14 in cotton fiber.

To put it simply, direct evidences are needed to annotate C3 cell cluster as cotton fiber cells. Without the direct evidences, I would say that this manuscript is providing the developmental knowledge on C3 cell cluster, but the cell type of this cluster remains unknown. I suggest the authors can perform either one of the following experiments to obtain a direct evidence: (1) Use a fiber cotton specifically expressed promoter driving GFP to isolate fiber cells; (2) Use laser capture microdissection to collect fiber cotton cells under microscope to anatomically ensure that cotton fiber cells are indeed collected. Both (1) and (2) can be followed by RNA-seq, and transcriptomic correlation can be performed along with the current cell clusters. The cell cluster with highest correlation would then can be identified as cotton fiber cells.

**Minor comments:**

Line1137, typo "elongation1"

**Reviewer 2**

**Were you able to assess all statistics in the manuscript, including the appropriateness of statistical tests used?**

Yes, and I have assessed the statistics in my report.

**Comments to author:**

This manuscript entitled "Cell-specific Clock Controlled Gene Expression Program Regulates Rhythmic Fiber Cell Growth in Cotton" uncovered fiber-specific circadian clock-controlled gene expression program in regulating cotton fiber growth. As far as I know, this is the first paper describing a cell atlas of cotton outer integument of ovules at the single-cell resolution and the authors provide valuable clue for the direct link between circadian rhythm and fiber growth. Overall, the topic is interesting and includes novel feature. Below I summarize a few non-crucial issues and puzzled points.

1. In L117, how to defined "-2/-1 DPA"? It is easy for understanding 0-2DPA which means the days after anthesis but it is hard to defined the time point of one/two days before flowering.

2. Font size in supplementary figure 2b is not conducive to reading.

3. In L213, references related to "its downstream-regulated gene MYB25" should be added.

4. In L223-225, this hypothesis is currently only an inference, and it is more suitable to be mentioned in the discussion and needs more evidence to support.

5. In Figure 3c, it can be seen that GhRALF1 (1 μM) can significantly inhibit fiber cell growth. Whether the influence of the concentration factor of RALF is excluded, the author can add a gradient concentration experiment to verify.

6. Additionally, in Figure 3d, there was a transient decrease in ECAR after injection with control which should be explained.

7. In Figure 4a-c, I found that the strength of the peak is related to the delay of the fiber growth rate, that is, the fastest growth rate of fiber occurs at 0-4h after the strong peak or 4-8h after the weak peak. Similarly, PH rise occurs at 0-4h after the strong peak or 4-8h after the weak peak. It might indicate that RALF1 promotes fiber growth rather than inhibits it. These also reflects the effect of transcript concentration on fiber growth.

8. The legends of some supplementary tables need to be added, e.g. supplementary table

9. And how did start and end of motifs identified and how many motifs are there? Please elaborate in the method.

9. In supplementary figure 8b-c, what does "small mad" and "big mad" mean? Please explain the calculation method of "random" in the figure legend and method.

10. In figure 5f and L409-411, you should specify what set of genes were used for enrichment tests. I would strongly advice using expressed genes in C3 and not all genes potentially regulated by TCP and TCP-like motifs.

11. In supplementary figure 8e, I still wonder the calculation method of "random" and error-bar.

12. In L381, the authors mentioned "117 and 95 genes with differentially chromatin accessibility", is there any cluster marker genes in it?

13. The methods and results of identification, filtering and classification of motifs are poorly readable and confusing. Can the authors provide a flow chart about the complete pipeline so that the reader can understand clearly.

**Author response:**

**Point-by-point responses:**

**Reviewer #1:**

**Major comments:**

Wang et al performed single cell RNA sequencing using the cells of Gossypium hirsutum outer integument (OI) of ovules from wildtype (WT) and fuzzless/lintless (fl) cotton. The authors annotated the cell type of C3 cell cluster as cotton fiber cells based on the following arguments: (1) Comparing the ovules in WT, the fl ovules exhibit deficiency on fiber initiation. Through the comparison of cell clusters between WT and fl, C3 cell cluster is absence in fl. (2) Some marker genes enriched with GO functional terms as trichome differentiation and initiation. (3) Some genes in C3 cell cluster are associated with cotton fiber. (4) Genes specifically expressed in cotton fiber cells.

Here are my comments toward these four arguments: For argument (1), the absence of C3 cell cluster in fl could be caused by different reasons. For example, fiber initiation may require a type of "supporting cells", and the failure of initiation may be caused by the absence of "supporting cells" instead of the absence of fiber cells. In addition, an obvious decrease of cells in C5 cluster also appeared in fl, which can also be annotated as fiber cotton cells or the unknown "supporting cells". For argument (2), the authors only selected the GO terms in C3 cluster that they favored for cell type annotation. Many other GO terms, instead, represent the majority and were not used for cell type annotation, such as organism reproduction, response to UV, root morphogenesis for transcription factors, and peptide biosynthetic process, cellular nitrogen compound biosynthetic process and amide biosynthetic process for non-transcription factors. For argument (3), genes associated with cotton fiber does not mean that these genes can serve as marker genes for cell type annotation. Take MYB25 as an example, the association of MYB25 and fiber cotton is illustrated by the regulation of MYB25 toward fiber development. However, numerous studies have shown that regulations of certain developmental processes can be exerted by non-cell-autonomous pathways. Namely, it is not necessary for a gene to be specifically expressed in cotton fiber cells to regulate their development. For argument (4), the authors seemed to misinterpret the conclusions from previous studies. Take a reference provided in the manuscript as an example, in Wang et al 2013, the following sentence was extracted: "Because it is technically unfeasible to separate the fiber initials from the ovules at early developmental stages, in situ hybridization was conducted to examine the expression of GhTCP14 in 0-DPA ovules when cotton fibers initiate from the ovule epidermal cells. GhTCP14 transcripts were also abundant in the fiber initials (Fig. 7B)." GhTCP14 was reported as an abundantly expressed gene not as a specifically expressed gene in cotton fiber, but this reference was used as a supporting evidence in this manuscript for specific gene expression of GhTCP14 in cotton fiber.

***Response****: Thanks for the comments. We now have provided direct evidence supporting that the cells in the C3 cluster are cotton fiber cells, as in the detailed response to the following question below.*

*We have corrected the original description “specifically expressed in fiber” of GhTCP14 as “fiber initials abundantly expressed transcription factor” and revised the main text as “Second, the genes in the C3 cluster include multiple known cotton fiber-associated transcription factors: MYB25 (Machado et al., 2009; Wan et al., 2016), GhHD1 (Walford et al., 2012), GhHOX3 (Shan et al., 2014); fiber initials abundantly expressed transcription factor GhTCP14 (Wang et al., 2013); and cotton fiber traits-associated genes from GWAS: GhTUA9 and qFWPB_D02 (He et al., 2021)”.*

To put it simply, direct evidences are needed to annotate C3 cell cluster as cotton fiber cells. Without the direct evidences, I would say that this manuscript is providing the developmental knowledge on C3 cell cluster, but the cell type of this cluster remains unknown. I suggest the authors can perform either one of the following experiments to obtain a direct evidence: (1) Use a fiber cotton specifically expressed promoter driving GFP to isolate fiber cells; (2) Use laser capture microdissection to collect fiber cotton cells under microscope to anatomically ensure that cotton fiber cells are indeed collected. Both (1) and (2) can be followed by RNA-seq, and transcriptomic correlation can be performed along with the current cell clusters. The cell cluster with highest correlation would then can be identified as cotton fiber cells.

***Response****: Thanks for the comment and great suggestions on the experiment and analysis. Similar to the suggested option (2), we have applied micromanipulation to capture morphologically visible fiber cells at 1 DPA on the epidermis of the ovule outer integument followed by single-cell RNA-seq. Moreover, we re-analyzed our recently published bulk RNA-seq data from manually separated fiber cells at 5 DPA (Wen et al., Science China Life Sciences, 2022). As described below, the results of both datasets now provide direct evidence supporting the annotation of the C3 cell cluster as cotton fiber cells.*

*For the captured fiber cells at 1 DPA, we performed SCAN-seq (Single cell amplification and sequencing of full-length RNAs by Nanopore platform) recently developed (Fan et al., PLOS Biology, 2020). In this assay, we captured five fiber cells from the outer integument (OI) of 1 DPA ovules from wildtype (WT) using micromanipulation (new Figure 1i). After qualification analysis of SCAN-seq data, we found two fiber cells have obtained more than 700,000 Nanopore long reads (new Supplementary Figure 4b). We then performed the suggested correlation analysis and the results show that the gene expression patterns of these two fiber cells (fiber1 & fiber3) have the highest correlation with the C3 cell cluster (new Supplementary Figure 4c). Furthermore, we performed expression pattern correlation analysis* *between the cells in the UMAP and the two fiber cells, and expectedly, the C3 cell cluster shows the highest correlations with two fiber cells (new Figure 4j).*

*We reanalyzed the bulk RNA-seq from three biological replicates of manually separated fiber cells at 5 DPA. The RNA-seq data of the three replicates show great reproducibility (new Supplementary Figure 4d). We next compared the gene expression patterns of these fiber cells with that of the five cell clusters (C1-C5), and we found that the gene expression correlation with the C3 cluster is the highest, followed by the C2 and C1 (new Supplementary Figure 4e). We also performed expression pattern correlation analysis between the cells in the UMAP and the three replicates of the collected fiber cells, and more significantly, the C3 cell cluster shows the highest correlations with all three replicates (new Figure 1k).*

*Collectively, based on the new direct evidence, we strongly believe that* *the cells of the C3 cell cluster are fiber cells. These new results, Figures 1i-k and Supplementary 4b-e shown below, have been incorporated into the revised manuscript.*

**New Figure 1i-k**. (i) A representative example illustrating the process of capturing individual fiber cells by micromanipulation under microscope. The fiber cell (white arrow) was dissociated from the epidermis of an ovule at 1 DPA and captured for single cell amplification and sequencing of full-length RNAs by Nanopore platform (SCAN-seq). Scale bar, 50 μm. (j-k) The gene expression correlation analysis between scRNA-seq and SCAN-seq of two individual fiber cells (j) and bulk RNA-seq of isolated fiber cells in three replicates (k). The Spearman correlation coefficients are shown for the cells in the UMAP of scRNA-seq from WT (top) and *fl* (bottom). The dotted line circle marks the C3 cell cluster from scRNA-seq.

**New Supplementary Figure 4b-e**. (b) Mapping statistics of SCAN-seq data for five fiber cells and two mock controls. The successfully sequenced libraries of cell 1 and cell 3 are shown in red. (c) The gene expression correlation analysis between the two individual fiber cells at 1 DPA (cell 1 and cell 3) from SCAN-seq and the cells in different clusters from scRNA-seq. (d) The data reproducibility analysis of the three replicates of bulk RNA-seq of isolated fiber cells at 5 DPA. (e) The gene expression correlation analysis between the fiber cells (5 DPA) from bulk RNA-seq and the cells in different clusters from scRNA-seq.

*Reference:*

*Fan, X., Tang, D., Liao, Y., Li, P., Zhang, Y., Wang, M., Liang, F., Wang, X., Gao, Y., Wen, L., Wang, D., Wang, Y., & Tang, F. (2020). Single-cell RNA-seq analysis of mouse preimplantation embryos by third-generation sequencing.* ***PLOS Biology****, 18(12), e3001017.*

*Wen, X., Zhai, Y., Zhang, L., Chen, Y., Zhu, Z., Chen, G., Wang, K., and Zhu, Y. (2022). Molecular studies of cellulose synthase supercomplex from cotton fiber reveal its unique biochemical properties.* ***Science China Life Sciences****, 65:9 65:1776–1793.*

**Minor comments:**

Line1137, typo "elongation1"

***Response****: Fixed as “elongation”.*

**Reviewer #2:**

This manuscript entitled "Cell-specific Clock Controlled Gene Expression Program Regulates Rhythmic Fiber Cell Growth in Cotton" uncovered fiber-specific circadian clock-controlled gene expression program in regulating cotton fiber growth. As far as I know, this is the first paper describing a cell atlas of cotton outer integument of ovules at the single-cell resolution and the authors provide valuable clue for the direct link between circadian rhythm and fiber growth. Overall, the topic is interesting and includes novel feature. Below I summarize a few non-crucial issues and puzzled points.

***Response****: We thank the reviewer for the positive comments and for affirming the innovativeness of our discoveries.*

1. In L117, how to defined "-2/-1 DPA"? It is easy for understanding 0-2DPA which means the days after anthesis but it is hard to defined the time point of one/two days before flowering.

***Response****: The cotton plants in this study are cultivated in the greenhouse with a steady growth environment, and the timing of the flowering and blossoming is stable. The flowers and bolls of cotton (Xuzhou 142 WT) at -2 to 2 DPA have distinct characteristics in appearance including the size of buds and the openness of the petals as shown in the new Supplementary Figure 1a. Thus, we can easily distinguish and collect ovules at two days and 1 day before flowering (-2 and -1 DPA) based on their unique features, well-accepted in the cotton field (Walford et al., Plant Journal, 2011; Chen et al., Nature Biotechnology, 2011).*

**New Supplementary Figure 1a.** (a) The flowers and bolls of cotton (Xuzhou 142 WT) at -3 to 2 DPA.

*Reference:*

*Walford, S.-A., Wu, Y., Llewellyn, D. J., and Dennis, E. S. (2011). GhMYB25-like: a key factor in early cotton fibre development.* ***Plant Journal****,65:785–797.*

*Chen, Z. J., & Guan, X. (2011). Auxin boost for cotton.* ***Nature Biotechnology****, 29(5), 407–409.*

2. Font size in supplementary figure 2b is not conducive to reading.

***Response****: We have increased the font size in Supplementary Figure 2b and adjusted the UMAP figure sizes to enhance the readability.*

3. In L213, references related to "its downstream-regulated gene MYB25" should be added.

***Response****: We apologize for missing the reference “Walford et al., 2011”, which has been added to the revised manuscript.*

4. In L223-225, this hypothesis is currently only an inference, and it is more suitable to be mentioned in the discussion and needs more evidence to support.

***Response****: Thanks for the comment. We have removed the original sentence of our hypothesis and pointed out that the factors upregulated in C2 including MYB25-like merit further studies in the revised manuscript.*

5. In Figure 3c, it can be seen that GhRALF1 (1 μM) can significantly inhibit fiber cell growth. Whether the influence of the concentration factor of RALF is excluded, the author can add a gradient concentration experiment to verify. 

***Response****: Thanks for the suggestion. We have added this gradient concentration experiment using 1 nM, 50 nM, 500 nM, and 1 μM of GhRALF1. The results showed that GhRALF1 could inhibit fiber cell growth in a concentration-dependent manner (new Supplementary Figure 5a), whereas GhRALF2 has a limited effect on fiber growth at different concentrations. These results have excluded the influence due to the concentration of RALF and we have added them to the revised manuscript.*

**New Supplementary Figure 5a.** (a) The effects on fiber growth of *in vitro* cultured ovules under GhRALF1 and GhRALF2 treatments at four different concentrations (1 nM, 50 nM, 500 nM, and 1 μM). Scale bar, 5 mm.

6. Additionally, in Figure 3d, there was a transient decrease in ECAR after injection with control which should be explained.

***Response****: Thanks for your suggestion. As the RALF peptides were dissolved with distilled water at pH 7.0, we used distilled water as the injection control. The transient decrease in ECAR after injection was due to the pH change due to the addition of distilled water. The slight pH increase (alkalinization of the originally weak acid environment) is sensed by the instrument and causes a decrease in the measured signals of the proton pump activity. We have added a brief explanation to the revised manuscript: “**We noted a transient decrease in ECAR after control injection, which was due to the pH change caused by the addition of distilled water”.*

7. In Figure 4a-c, I found that the strength of the peak is related to the delay of the fiber growth rate, that is, the fastest growth rate of fiber occurs at 0-4h after the strong peak or 4-8h after the weak peak. Similarly, PH rise occurs at 0-4h after the strong peak or 4-8h after the weak peak. It might indicate that RALF1 promotes fiber growth rather than inhibits it. These also reflects the effect of transcript concentration on fiber growth.

***Response****: Thanks for your hypothesis. As you proposed, we could not exclude the possibility that in vivo physiological role of RALF1 for cotton fiber growth may be activation rather than inhibition, although we found that the RALF1 inhibits fiber growth in vitro. A recent study in Arabidopsis also supported that the RALF1 homolog triggers root growth inhibition (Li et al., PNAS, 2022). In the original manuscript, we noted the 4h delay and ascribed the delay to the time needed for the increased RALF1 mRNA transcripts to be translated to peptide and its secretion. In the revised manuscript, we have emphasized that the RALF1’s inhibitory effect is based on in vitro experiments, and included another interpretation that GhRALF1 might promote fiber cell elongation in vivo. We pointed out that the physiological effect in vivo remains to be elucidated by further studies.*

*Reference:*

*Li L, Chen H, Alotaibi SS, Pěnčík A, Adamowski M, Novák O, Friml J. RALF1 peptide triggers biphasic root growth inhibition upstream of auxin biosynthesis.* ***Proc Natl Acad Sci USA****, 2022, 119(31), e2121058119.*

8. The legends of some supplementary tables need to be added, e.g. supplementary table 9. And how did start and end of motifs identified and how many motifs are there? Please elaborate in the method.

***Response****: We apologize for missing details. We have included legend details for all supplementary tables. As in the revised Supplementary Table 9, we added a cover description sheet to summarize the contents and describe the columns of all sheets. The start and end of the motifs represent the positions of corresponding 8-mer motifs in the genome. There are 317 8-mers and we have elaborated the description in the Methods of the revised manuscript.*

9. In supplementary figure 8b-c, what does "small mad" and "big mad" mean? Please explain the calculation method of "random" in the figure legend and method.

***Response****: The MAD standards for Median Absolute Deviation (MAD) of the relative distance to TSS. For a certain cell-specific k-mer motif, we computed the MAD for its forward motif (MAD_forward_) and reverse motif (MAD_reverse_). The “small mad” and “big mad” in the original manuscript represent the smaller one and the bigger one between the two MAD_forward_ and MAD_reverse_ values, which are min(MAD_forward_, MAD_reverse_) and max(MAD_forward_, MAD_reverse_), respectively. The “random” represents results from randomization control experiments, in which we shuffled the same number of k-mer motifs in ACRs and repeated it 100 times. The distribution of the relative distance of randomly shuffled k-mer motifs was labeled as “random”. We have provided a flow chart to illustrate this procedure in the new Supplementary Figure 8c, revised the labels for Supplementary Figure 8d-e (original 8b-c), and added the description in the Methods.*

New Supplementary Figure 8c. Flowchart showing the procedure for identifying direction-specific motifs.

10. In figure 5f and L409-411, you should specify what set of genes were used for enrichment tests. I would strongly advice using expressed genes in C3 and not all genes potentially regulated by TCP and TCP-like motifs.

***Response****: Thanks for the suggestion. We indeed used the expressed genes in C3 which were potentially regulated by TCP and TCP-like motifs in the enrichment analysis. We have specified it in the legend of Figure 5f and revised the original sentence to “the C3 expressed genes potentially regulated by TCP and TCP-like motifs are significantly enriched in the biological processes of protein translation and mitochondrion”.*

11. In supplementary figure 8e, I still wonder the calculation method of "random" and error-bar.

***Response****: Briefly, we* *randomly selected the same number of genes as the TCP/TCP-like motif target genes 10,000 times and counted their highest expressed cell types.* *The mean value and standard deviation of the 10,000 replicates were used as the random value and the error bar for the random sample. We have added the description in the legend of Supplementary Figure 8g (original 8e) and in the Methods.*

12. In L381, the authors mentioned "117 and 95 genes with differentially chromatin accessibility", is there any cluster marker genes in it?

***Response****: We examined the relationship between cluster marker genes with those differentially accessible regions (DARs) and the genes with differential chromatin accessibility (DAGs) based on summarized signals from active chromatin regions in the gene body and promoter regions. We found that there was no marker gene among those DAGs, and there were a small number of 23 marker genes harboring DAR(s), consistent with the chromatin heterogeneity* *being limited* *among cell clusters from the OI of the ovules. We have included the data in the revised Supplementary Table 9 and added the results to the revised manuscript.*

13. The methods and results of identification, filtering and classification of motifs are poorly readable and confusing. Can the authors provide a flow chart about the complete pipeline so that the reader can understand clearly.

***Response****: Thanks for your suggestion. We have provided a flow chart of the complete pipeline (new Supplementary Figure 8a) and also added detailed descriptions in the Methods section of the revised manuscript.*

**New Supplementary figure 8a**. Flowchart showing the computational pipeline for identifying cell-specific motifs.

**Second round of review**

**Reviewer reports:**

**Reviewer #1:**

The authors have provided some evidences to support their annotation on cotton fibers using two datasets as the micromanipulation for fiber cell capture and the re-analyses of their previous fiber bulk RNA-seq. Noting that the analysis results from both datasets showed high correlation to C3 cluster, however, the fact that C3 cluster representing cotton fibers still remained as a questionable task to be addressed. In addition, the authors did not address all concerns raised in my previous comments.

Major comments

(1) The authors agreed that GhTCP14 is not specifically expressed in cotton fiber cells, then this gene can no longer be used as a marker gene. Since the misinterpretation in the original manuscript on the results from previous studies, I suggest the authors to provide the descriptions from the previous studies in Response to Reviewer to report the other genes as specifically expressed genes.

(2) The authors did not respond to my previous concerns related to GO terms. “The authors only selected the GO terms in C3 cluster that they favored for cell type annotation. Many other GO terms, instead, represent the majority and were not used for cell type annotation, such as organism reproduction, response to UV, root morphogenesis for transcription factors, and peptide biosynthetic process, cellular nitrogen compound biosynthetic process and amide biosynthetic process for non-transcription factors.

(3) In the figures of 30- and 60-min digestion of Figure 1i, only cloudy and black shadow could be observed, which is difficult to ensure the identity of the released cells as cotton fiber. On the right part of Figure 1i, the diameter of the glass microneedle is much larger than the cell. Besides the cell pointed by the arrow, many other particles in this figure could also be cells with unknown identities. With the description in Methods for cell capture as “sucked at the tip of a glass microneedle”, the contamination on the capture of multiple cell types could be highly possible.

(4) In Figure 1j, the variation between cell 1 and 3 is very high, leading to the concern of the experimental robustness.

(5) The authors only used two cell (cell 1 and 3, Figure 1j) for the cell type annotation, which is not convincing.

(6) The correlation of cell 1 and cell 3 (Figure 1j) with the single-cell transcriptional profile are relatively high in cluster 3. However, parts of cluster 1, 2 and 4 also show high correlation with cell 1 and cell 3 in WT. Especially in cell 1, the correlation of single-cell transcriptional profile with cluster 3 is almost similar to that with top left area of cluster 4. The results brought up another concern: Are the number of cell clusters sufficient to represent the cell types generated from the single-cell transcriptional results? Different area in cluster 4 exhibit very different correlations to cell 1 and cell 3.

(7) The authors described that they re-analyzed their published bulk RNA-seq data from manually separated fiber cells at 5 DPA (Wen et al., Science China Life Sciences, 2022). Only one sentence was found to be related to fiber cell collection in the MATERIALS AND METHODS of this article “fiber cells were carefully separated from the epidermis of cotton ovules with tweezers on ice at 5, 10, 15, 20, 25, and 30 DPA”. However, this description is not sufficient to demonstrate the collection of cotton fibers without any other cell type contamination.

**Reviewer #2:**

The authors have addressed most of my concerns. I have no further comment.

**Author response:**

**Point-by-point responses:**

**Reviewer #1:**

The authors have provided some evidences to support their annotation on cotton fibers using two datasets as the micromanipulation for fiber cell capture and the re-analyses of their previous fiber bulk RNA-seq. Noting that the analysis results from both datasets showed high correlation to C3 cluster, however, the fact that C3 cluster representing cotton fibers still remained as a questionable task to be addressed. In addition, the authors did not address all concerns raised in my previous comments.

***Response:*** *Thanks for the comments and suggestions. Briefly, in the revision, we have taken your suggestion and used laser-capture microdissection (LCM) to harvest fiber cells from the ovule sections and performed NGS RNA sequencing for gene expression analysis. The new LCM-seq data for fiber cells show good reproducibility between three replicates and have the highest correlation with the C3 cell cluster, which is convincing to conclude that the C3 cluster represents cotton fibers (see the responses to your major comment 4). We have addressed all the concerns including a complete description of GO-terms missing in the last revision. We hope that you are satisfied with this revision.*

**Major comments:**

(1) The authors agreed that GhTCP14 is not specifically expressed in cotton fiber cells, then this gene can no longer be used as a marker gene. Since the misinterpretation in the original manuscript on the results from previous studies, I suggest the authors to provide the descriptions from the previous studies in Response to Reviewer to report the other genes as specifically expressed genes.

***Response:*** *Thanks for the suggestion. We have carefully reviewed 11 fiber-associated genes from previous studies, among which, there are 4, 5, and 2 genes with experimental evidence from in situ hybridization, GUS reporter, and qRT-PCR assays, respectively. Their original descriptions and corresponding experimental evidence are summarized below and in* ***Figure R1****.*

*The following 5 fiber-associated genes were identified as marker genes in* ***Supplementary Table 2*** *of our manuscript. The* *original descriptions in previous studies have been extracted and listed below.*

***GhTCP14:*** *Wang et al., 2013. Plant Physiol. doi:10.1104/pp.113.215673. In Main Text: “…in situ hybridization was conducted to examine the expression of GhTCP14 in 0-DPA ovules…GhTCP14 transcripts were also* ***abundant in the fiber initials****”. In Discussion: “In this work, we found that GhTCP14 is* ***specifically expressed at*** *the fiber initiation and elongation stages...”.*

***GhMYB109****: Suo et al., 2003. Biochim Biophys Acta. doi:10.1016/j.bbaexp.2003.08.009.* *“...GhMYB109 was shown to be* ***specifically expressed in*** *fiber initials and elongated fibers, suggesting that it likely plays a role in the initiation and elongation of cotton fiber.”.*

***GhTUA9****: Li et al., 2007. J Exp Bot. doi:10.1093/jxb/erm167. “...****strong GUS activity was detected exclusively in*** *the most of fibres, while no or weak GUS staining was observed in ovules.”.*

***GhMYB25****: Machado et al., 2009. Plant J. doi:10.1111/j.1365-313X.2009.03847.x.*

*“Strong GUS activity was detected in 0 dpa ovules and localized in thin sections to the ovule epidermis, showing* ***much higher expression in*** *the expanding fibre initials and the elongating fibres at 10 dpa. ”.*

***GaHOX3/GhHOX3****: Wang et al., 2004. Plant Cell. doi:10.1105/tpc.104.024844. “Real-time PCR showed that GaMYB2 transcripts were indeed* ***highly enriched in*** *developing fiber cells, and the expression level was either rather low or undetectable in all other tissues examined. A similar expression pattern was observed for the HOX3 and RDL1 genes…”.*

*The following 6 fiber-associated genes were identified as highly expressed genes in* ***Supplementary Table 2*** *of our manuscript. Their original descriptions in previous studies have been extracted and listed below.*

***GaMYB2/GhMYB2****: Wang et al., 2004. Plant Cell. doi:10.1105/tpc.104.024844. “In situ mRNA hybridization of 0-DPA ovules* ***localized the GaMYB2 transcripts in fiber initials****, confirming its fiber-specific expression in ovules.”.*

***GhABP19/20****: Ji et al., 2003. Nucleic Acids Res. doi:10.1093/nar/gkg358. “...strong hybridization was* ***localized mainly in*** *elongating fiber cells...Similar results were obtained when sections were probed with either the ABP19/20 homolog...”.*

***GhHD-1****: Walford et al., 2012. Plant J. doi:10.1111/j.1365-313X.2012.05003.x. “Our reporter studies have shown that GhHD-1is also mostly epidermal specific …, and then* ***more strongly in*** *the cells developing into fibres as well as in some other tissues in epidermal layers and trichomes …”.*

***GbPDF1/GhPDF1****: Deng et al., 2012. Plant Physiol. doi:10.1104/pp.111.186742. “...we found that the transcripts of GbPDF1* ***accumulated predominantly in*** *the fibers, especially at the early stage, suggesting its potential role in fiber development...”.*

***GhACTIN1****: Li et al., 2005. Plant Cell. doi:10.1105/tpc.104.029629. “...****strong GUS activity was detected only in*** *young fibers, whereas no or weak GUS staining was observed in ovules, anthers, petals, sepals, leaves, and roots, including their trichomes.”.*

***GhFLA1****: Huang et al., 2008. Physiol Plant. doi:10.1111/j.1399-3054.2008.01139.x.*

*“Among the 19 GhFLA genes, GhFLA1 and GhFLA4 were* ***specifically expressed in*** *fibers.”*

*In our study, a marker gene for any cluster such as the C3 cluster was identified by a differential gene expression analysis requiring its expression in the C3 cluster to be at least 2-fold higher than that in any other clusters with a statistical significance FDR<1e-9 using the findmarker functions in scran package with its default thresholds (see the details in* ***Materials and Methods****). Actually, this is a widely adopted method for selecting marker genes in scRNA-seq data analysis, such as the findmarker function in scran or FindMarkers function in seurat tools [Haghverdi et al., 2018; Trapnell et al., 2014]. For our scRNA-seq data, we identified 339 marker genes for the C3 cluster (****Figure 1e*** *and* ***Supplementary Table 2)****. Those non-marker* *genes showing the highest expression in the C3 cluster were defined as C3 highly expressed genes.*

**Figure R1.** **The fiber-associated genes from the literature and their expression from scRNA-seq in this study.** (**a**) The references and experimental evidence from previous studies for the five marker genes in the C3 cluster. (**b**) Single-cell UMAP profiles (top) and quantified gene expression values (CPM) in C1-C5 clusters (bottom) for the five marker genes in the C3 cluster. (**c**) The references and experimental evidence from previous studies for the six highly expressed genes in the C3 cluster. (**d**) Single-cell UMAP profiles (top) and quantified gene expression values (CPM) in C1-C5 clusters (bottom) for the six highly expressed genes in the C3 cluster. The cells in the C3 cluster are marked with dotted line circles. The experiment methods in previous studies are annotated below the original figures, including RNA *in situ* hybridization, GUS reporter, and qRT-PCR.

*According to our criteria, the 11 fiber-associated genes are identified as five marker genes and six highly expressed genes for the C3 cluster. As you can see, their expressions are highly enriched in the C3 cluster,* *the fiber cells that we annotated according to our scRNA-seq data (****Figure R1b,d****), consistent with these previous studies (****Figure R1a,c****). These results indicate* *that our marker genes for C3 fiber cells are reliable.*

*Of note, we identified “marker genes” and “highly expressed genes” such as for the C3 cluster based on their relative expressions among the five clusters, not requiring “specifical” or “unique” expression in the C3 cluster. The expression level of GhTCP14 met the criterion of marker gene and thus it is defined as one of the 349 marker genes for the C3 cluster.*

*We think that these results could be taken as* *an important piece of evidence to support the conclusion that the cells in the C3 cluster are fiber cells. We have added* ***Figure R1*** *as the new* ***Supplementary Figure 5*** *in the revised manuscript.*

***References:***

*Haghverdi L, Lun ATL, Morgan MD, Marioni JC.* *Batch effects in single-cell RNA-sequencing data are corrected by matching mutual nearest neighbors. Nat Biotechnol. 2018;36:421–7.*

*Trapnell C, Cacchiarelli D, Grimsby J, Pokharel P, Li S, Morse M, et al.* *The dynamics and regulators of cell fate decisions are revealed by pseudotemporal ordering of single cells. Nat Biotechnol. 2014;32:381–6.*

(2) The authors did not respond to my previous concerns related to GO terms. “The authors only selected the GO terms in C3 cluster that they favored for cell type annotation. Many other GO terms, instead, represent the majority and were not used for cell type annotation, such as organism reproduction, response to UV, root morphogenesis for transcription factors, and peptide biosynthetic process, cellular nitrogen compound biosynthetic process and amide biosynthetic process for non-transcription factors.”

***Response:*** *We apologize for missing to respond the concern in the last revision. We further re-analyze these major GO-terms (Biological Process, BP) for transcription factors (TFs) and non-TFs groups.*

*For the top 10 GO-terms that you mentioned for the non-TFs group* *(****Figure R2a*** *left), the hierarchical relationship is visualized in* ***Figure R2b****, and they are associated with 167 marker genes in the C3 cluster, 145 of which are shared by these 10 GO-terms (****Figure R2c****). Combining these two results, we find that the TOP 10 GO-terms enriched in non-TFs for the C3 cluster mainly focus on the process of “protein translation (GO:0006412)”, the bottommost GO-term in the dendrogram* *of* ***Figure R2b****. Additionally, it is important to note that nearly all the 167 genes are potentially regulated by TCPs (TCP and TCP-like) genes* *(****Figure R2c****) according to our previous analysis results (****Figure 5e-f****,* ***Supplementary Figure 9f,*** *and* ***Supplementary Table 11)****. This indicates that most of these enriched genes are regulated by the highly expressed TCPs TFs in fiber cells,* *supporting that the cells in the C3 cluster are fiber cells.*

**Figure R2.** **The analysis for the top 10 GO-terms and associated genes of C3 cluster marker genes.** (**a**) The top 10 enriched GO-terms for the marker genes in the C3 cell cluster. Left: non-transcription factors (non-TFs); right: Transcription factors (TFs). (**b**) Hierarchical relationships for the top 10 GO-terms of the non-TFs group. (**c**) The association between non-TFs GO-terms and 167 marker genes. The right panel shows their putative regulations by TCP/TCP-like TFs with black squares indicating the genes contain TCP/TCP-like binding motifs in their promoters. (**d**) Hierarchical relationships for the top 8 GO-terms of TFs group. (**e**) The association between the top 10 TFs GO-terms and 15 marker genes in the C3 cell cluster.

*For the top 10 GO-terms for the TFs group (****Figure R2a*** *right), 8 of them have direct hierarchical relationships as shown in* ***Figure R2d****, and there are 15 associated marker genes in the C3 cluster* *(****Figure R2e****). As you can see, the “multicellular organism reproduction (GO:0032504)” and its child term “cellular process involved in reproduction in multicellular organism (GO:0022412)” connect to other terms in the node of “seed trichome differentiation (GO:0090376)”* *(****Figure R2d****), and share the same associated genes with “seed development (GO:0048316)”* *(****Figure R2e****). Moreover, it should be pointed out that the associated genes for “response to UV (GO:0009411)” and “root morphogenesis (GO:0010015)” (marked in red in* ***Figure R2e****) are actually contained in the gene list associated with the other 8 GO-terms, explaining the enrichment of the two terms.*

*Together, the results from our in-depth analysis of the GO-terms show that the top GO-terms enriched in the TFs group are directly linked to the cell differentiation and development of trichome or seed, and related processes, and also show that the majority of top GO-terms enriched in the non-TFs group are mainly associated with protein translation and are regulated by TCP/TCP-like TFs activated in fiber development. These results also support that the cells in the C3 cluster are fibers.*

(3) In the figures of 30- and 60-min digestion of Figure 1i, only cloudy and black shadow could be observed, which is difficult to ensure the identity of the released cells as cotton fiber. On the right part of Figure 1i, the diameter of the glass microneedle is much larger than the cell. Besides the cell pointed by the arrow, many other particles in this figure could also be cells with unknown identities. With the description in Methods for cell capture as “sucked at the tip of a glass microneedle”, the contamination on the capture of multiple cell types could be highly possible.

***Response:*** *When we perform enzyme digestion and track the real-time dissociation of fiber cells under the light field of a microscope, the fiber cells on* *the surface of the ovules can be seen clearly, although the main body of the intact ovules exhibits cloudy and black shadow (****Figure R3a*** *top). When we use the ovules of mutant fl at 1 DPA to take images, there are no fiber cells in the marginal part of the shadow under the light field (****Figure R3a*** *bottom). This enables us to track released fiber cells from the surfaces of ovules.*

**Figure R3. The micromanipulation of cotton fiber cells.** (**a**) Ovules and fiber cells under the light field of the microscope. The fiber cells in WT are marked with white arrows (top) and they are not available in *fl*. (**b**) The schematic view of isolating a single fiber cell under a microscope including a series of dilutions with 8% mannitol.

*Although the diameter of the glass microneedle is larger than the cell, we have several continuous purification steps after the first capture of released fiber cells from the enzyme-digested solution, which are tracked in real time under the microscope (****Figure R3b****). These* *purification steps including gradient dilution and mannitol wash ensure that one fiber cell (rod-shaped cell) without other cells and cell residues is retained to carry out the following single-cell library construction. Although we replaced these data with LCM-seq data (****Figure R4*** *below), we believe that the identity of fiber cells can be ensured and the possibility of contamination should be rare in SCAN-seq.*

(4) In Figure 1j, the variation between cell 1 and 3 is very high, leading to the concern of the experimental robustness.

***Response:*** *Thanks for pointing out this concern. The SCAN-seq protocol uses one cell to construct a full-length RNA sequence library followed by Nanopore long-reads sequencing. The high variation may be due to the ultra-low quantity of RNA in one cell and the difficulty in RNA library construction, which are inherent problems of this technology. As described below, we tried the strategy of laser-capture microdissection (LCM) as you initially suggested, and found that LCM-seq results show very good robustness.*

*We developed LCM-seq by using laser-capture microdissection to harvest fiber cells coupled with NGS RNA sequencing for gene expression analysis. Briefly, we capture single fiber cells using LCM (Figure R4a), and pool 30 fiber cells for RNA sequencing as one replicate. We performed three replicates of LCM-seq in this revision. The sequencing data from the three biological replicates exhibit very good reproducibility (Figure R4b). Most importantly, the expression correlation analysis shows that the gene expression profiles of fiber cells have the highest correlation with the C3 cell cluster (Figure R4c-d). The new results support that the C3 cluster represents cotton fiber cells.*

*Thus, we replaced the SCAN-seq data with our new LCM-seq results in the revised manuscript. These results of LCM-seq have been integrated into* ***Figure 1i-j*** *and* ***supplementary Figure 3b-c****. The detailed method for LCM-seq, as described below, has been added in the* ***Materials and Methods*** *section of the revised manuscript. The sequencing data of LCM-seq have been deposited into the NCBI SRA database under project PRJNA847210.*

**Figure R4. LCM-seq of fiber cells at 1 DPA.** (**a**) A representative example illustrating the process of capturing fiber cells by laser-capture microdissection (LCM). The fiber cells indicated by star symbols were cut from the epidermis of an ovule at 1 DPA. Scale bar, 50 μm. (**b**) The data reproducibility analysis of the three replicates of LCM-seq of fiber cells at 1 DPA. (**c**) The gene expression correlation analysis between the fiber cells at 1 DPA from LCM-seq and the cells in different clusters from scRNA-seq. (**d**) The gene expression correlation analysis between scRNA-seq and LCM-seq of 1 DPA fiber cells in three replicates. The Spearman correlation coefficients are shown for the cells in the UMAP of scRNA-seq from WT (top) and fl (bottom). The dotted line circle marks the C3 cell cluster from scRNA-seq.

***Laser-capture microdissection and RNA sequencing (LCM-seq)***

*The* *laser-capture microdissection* *for fiber cells from cotton ovule sections is referred to a* *previous study [Wu et al., 2007]. The cotton ovules at 1DPA were embedded in Tissue-Tek® OCT (Sakura, Tokyo, Japan), frozen immediately in liquid nitrogen, then cryosectioned. A 10 μm thickness was chosen to keep monolayer cells in the z-axis as much as possible for each section, which minimized the contaminations from the cells in the z-axis for thick sections. The fiber cells were individually harvested by laser-capture microdissection with cryomicrotome (Leica, Germany),* *and lysed in 0.2% Triton X-100. A total of 30 fiber cells were pooled as one replicate for sequencing. Three biological replicates were prepared. The low-input mRNA-Amplification Kit (N712, Vazyme, China) and TruePrep® DNA Library Prep Kit (TD503, Vazyme, China) were used in the RNA sequencing library construction. The libraries were sequenced on* *DNBseq-T7 (BGI, China) with PE100 mode.*

***References:***

*Wu Y, Llewellyn DJ, White R, Ruggiero K, Al-Ghazi Y, Dennis ES. Laser capture microdissection and cDNA microarrays used to generate gene expression profiles of the rapidly expanding fibre initial cells on the surface of cotton ovules. Planta. 2007 Nov;226(6):1475-90. doi: 10.1007/s00425-007-0580-5.*

(5) The authors only used two cell (cell 1 and 3, Figure 1j) for the cell type annotation, which is not convincing.

***Response:*** *As described above, we have replaced SCAN-seq data with three biological replicates of LCM-seq data, the results of which are convincing to support that the C3 cluster represents cotton fiber cells.*

(6) The correlation of cell 1 and cell 3 (Figure 1j) with the single-cell transcriptional profile are relatively high in cluster 3. However, parts of cluster 1, 2 and 4 also show high correlation with cell 1 and cell 3 in WT. Especially in cell 1, the correlation of single-cell transcriptional profile with cluster 3 is almost similar to that with top left area of cluster 4. The results brought up another concern: Are the number of cell clusters sufficient to represent the cell types generated from the single-cell transcriptional results? Different area in cluster 4 exhibit very different correlations to cell 1 and cell 3.

***Response:*** *Thanks for your careful interpretation and suggestions. As described above in response to major comment 4, SCAN-seq data has high variation and low robustness due to its technical difficulties. In this revision, we used LCM-seq as you suggested, and the new data from LCM-seq show good consistency in correlation analysis. The cells in the C3 cluster are most strongly correlated with fiber cells from LCM-seq followed by the C2 cluster with a weaker correlation, consistent with the developmental trajectory from C2 to C3 (****Figure R4c****). We thus replaced the SCAN-seq data added in the last revision with the new LCM-seq data in the revised manuscript.*

*For another concern you raised, we agree that the cells in cluster C4 might be not sufficiently classified. In our analysis, cluster C4 could be further classified into five subgroups in the mode of over-clustering (****Supplementary Figure 2c-d****). In the current study, we mainly focus on the fiber cell cluster identified by comparing WT and its non-fiber mutant fl, with further experimental validation and characterization. Detailed dissection of other clusters including C4 and C5 merits further studies in the future.*

(7) The authors described that they re-analyzed their published bulk RNA-seq data from manually separated fiber cells at 5 DPA (Wen et al., Science China Life Sciences, 2022). Only one sentence was found to be related to fiber cell collection in the MATERIALS AND METHODS of this article “fiber cells were carefully separated from the epidermis of cotton ovules with tweezers on ice at 5, 10, 15, 20, 25, and 30 DPA”. However, this description is not sufficient to demonstrate the collection of cotton fibers without any other cell type contamination.

***Response:*** *Sorry for missing detailed information on sample preparation for published bulk RNA-seq data. For fiber sample collection, the cotton ovules at 5-30 DPA were plucked with a scalpel from the cotton bolls at the corresponding developmental stage. Then the fibers on the epidermis of the ovule were pulled out by two tweezers: one clamping the ovule and the other pulling a small bunch of fibers by pinching the tip of these fibers. Considering the fiber cells are much larger than the other epidermal cells, we believe the contaminated cells in the manually separated fiber cells would be very limited.*

**Reviewer #2:**

The authors have addressed most of my concerns. I have no further comment.

***Response:*** *We are happy to see that our last revisions have fully addressed the concerns of this Reviewer. We thank Reviewer #2 for his or her comments to strengthen our work. In this revision, we have used laser-capture microdissection coupled with RNA-seq (LCM-seq) to profile the gene expression of fiber cells. The new data obtained from LCM-seq are highly reproducible and show the highest correlation with the C3 cell cluster, further supporting that the C3 cluster represents cotton fibers. Since SCAN-seq data have high variation, we have replaced the SCAN-seq data added in the last revision with better LCM-seq results in the revised manuscript. The revisions are not relevant to the concerns of this Reviewer.*

**Third round of review**

**Reviewer reports:**

**Reviewer #1:**

In this round of review, the responses from the authors are very impressive. I have no further concerns or comments.
